# Sex- and Development-Dependent Responses of Rat Microglia to Pro- and Anti-inflammatory Stimulation

**DOI:** 10.3389/fncel.2018.00433

**Published:** 2018-11-20

**Authors:** Starlee Lively, Raymond Wong, Doris Lam, Lyanne C. Schlichter

**Affiliations:** ^1^Division of Genetics & Development, Krembil Research Institute, University Health Network, Toronto, ON, Canada; ^2^Department of Physiology, University of Toronto, Toronto, ON, Canada

**Keywords:** microglial activation, brain development, IFNγ plus TNFα, transcription profiling, IL-4, sex, female, ion channels

## Abstract

Addressing potential sex differences in pre-clinical studies is crucial for developing therapeutic interventions. Although sex differences have been reported in epidemiological studies and from clinical experience, most pre-clinical studies of neuroinflammation use male rodents; however, sexual dimorphisms in microglia might affect the CNS inflammatory response. Developmental changes are also important and, in rodents, there is a critical period of sexual brain differentiation in the first 3 weeks after birth. We compared rat microglia from sex-segregated neonates (P1) and at about the time of weaning (P21). To study transitions from a basal homeostatic state (untreated), microglia were subjected to a pro-inflammatory (IFNγ + TNFα) or anti-inflammatory (IL-4) stimulus. Responses were compared by quantifying changes in nitric oxide production, migration, and expression of nearly 70 genes, including inflammatory mediators and receptors, inflammasome molecules, immune modulators, and genes that regulate microglial physiological functions. No sex differences were seen in transcriptional responses in either age group but the IL-4-evoked migration increase was larger in male cells at both ages. Protein changes for the hallmark molecules, NOS2, COX-2, PYK2 and CD206 correlated with mRNA changes. P1 and P21 microglia showed substantial differences, including expression of genes related to developmental roles. That is, P21 microglia had a more mature phenotype, with higher basal and stimulated levels of many inflammatory genes, while P1 cells had higher expression of phagocytosis-related molecules. Nevertheless, cells of both ages responded to IL-4 and IFNγ + TNFα. We examined the Kv1.3 potassium channel (a potential target for modulating neuroinflammation) and the Kir2.1 channel, which regulate several microglia functions. Kv1.3 mRNA (*Kcna3*) was higher at P21 under all conditions and male P21 cells had higher mRNA and Kv currents in response to IFNγ + TNFα. Overall, numerous transcriptional and functional responses of microglia changed during the first 3 weeks after birth but few sex-dependent changes were seen.

## Introduction

Increasingly, it is recognized that sex can profoundly influence both the susceptibility and response to disease (Ober et al., 2008; Regitz-Zagrosek, 2012). Consequently, funding and government agencies have begun to mandate that both females and males be used in pre-clinical and clinical studies. Concerns about sexual dimorphisms extend to the central nervous system (CNS), where disparities are seen in the prevalence, severity, and outcomes of several diseases and disorders (Bilbo, 2018). Innate and adaptive immune responses contribute to the pathogenesis and severity of many CNS disorders (Shabab et al., 2017; Skaper et al., 2018) and there is growing evidence for sex differences in inflammatory responses both peripherally and in the CNS (Hanamsagar and Bilbo, 2016; Klein and Flanagan, 2016).

Sex differences arise from both chromosomal and hormonal influences. Genes on the sex chromosomes play essential roles in sex differentiation of the CNS, especially the male-inducing SRY gene (Manoli and Tollkuhn, 2018). Also, the X chromosome has a disproportionately high number of immune-related genes (Bianchi et al., 2012). In females, Barr body formation silences alleles on the extra X chromosome to prevent gene over-expression (Berletch et al., 2011) but genes escaping this inactivation might also confer sex-dependent differences. Early developmental and hormonal changes can also contribute to sex differences in the brain. Male rodents experience a surge in testosterone shortly before birth, which dissipates within a few hours and then sex differences begin to emerge. The testosterone surge influences development of neural cells and plays important organizational roles in establishing sexual dimorphisms in neural circuitry, notably in areas responsible for sexually divergent behaviors (Clarkson and Herbison, 2016). CNS changes include a transient enlargement of the parietal cortex, hippocampus and amygdala in males, and a transient increase in the number of microglia in these regions (Schwarz et al., 2012). Not enough is known about whether there are sex differences in responses of individual CNS cell types. For instance, both genetic differences and early exposure to sex hormones have the potential to alter microglial responses to inflammatory stimuli, and it is possible that such changes are sustained after they are removed from the brain.

Microglia react to CNS damage by acquiring characteristics conducive to dealing with altered homeostasis (Colonna and Butovsky, 2017; Li and Barres, 2018). Responses include receptor-mediated signaling that can change their morphology, migratory and phagocytic capacities, proliferation, expression of intracellular enzymes and antigen-presenting surface receptors, and release of pro- and anti-inflammatory molecules into the extracellular environment (Kabba et al., 2018; Li and Barres, 2018). A pro-inflammatory phenotype can be induced in neonatal microglia using a combination of interferon-γ (IFNg) and tumor necrosis factor-α (TNFα) (IFNγ + TNFα; ‘I+T’) *in vitro* (Spanaus et al., 1998; Lam et al., 2017). As recently summarized (Lively and Schlichter, 2018), both cytokines are rapidly up-regulated after acute CNS damage and are especially relevant in the absence of pathogenic organisms. Isolated microglia can also be skewed to various anti-inflammatory states by interleukin (IL)-4, IL-10, transforming growth factor β1 (TGFβ1) or glucocorticoids, and then they release immune mediators that promote scavenging, inflammation resolution, and repair (Kabba et al., 2018).

Microglial responses are routinely studied *in vitro* but most often using cells isolated from neonatal pups. Based on intrinsic differences at the chromosome level and early developmental and hormonal changes, we compared neonatal (P1) and prepubertal (P21) rat microglia and asked whether they show sexually dimorphic responses to I+T and IL-4. These ages were chosen for several reasons. First, microglia have very different morphologies and functions at these times. At birth, microglia are amoeboid, with or without short processes; and they are highly migratory and phagocytic, actively engulfing apoptotic neurons (Colonna and Butovsky, 2017; Li and Barres, 2018). Second, by P20 (just before weaning), the rat cortex has reached 90% of its final weight, neurons have migrated to their appropriate positions, synaptogenesis and myelination are well underway, and intriguingly, the regenerative resiliency observed in younger animals is lost (Semple et al., 2013). Third, by P20, rodent microglia are more ramified, and the main homeostatic functions are surveillance, synaptic pruning, and promoting oligodendrocyte progenitor cell survival and differentiation (Colonna and Butovsky, 2017; Li and Barres, 2018).

To assess the initial state and stimulus-evoked responses, we quantified transcript expression of a wide range of inflammatory mediators, immune receptors and modulators; and molecules related to microglia physiological functions, including phagocytosis and production of reactive oxygen species. Although microglia of both sexes and ages showed many transcriptional changes in response to I+T and IL-4; no sex differences were apparent before or after stimulation. In the age comparison (P1 versus P21), we found substantial differences in gene expression, with or without inflammatory stimuli. Last, we examined expression of two K^+^ channels (Kv1.3/*Kcna3*, Kir2.1/*Kcnj2*) and the Kv and Kir currents (at P21), which are known to regulate some microglial functions that are important during the first 3 weeks after birth. The only sex difference was that the I+T-induced increase in Kv current in P21 microglia was higher in males. The only age difference was that *Kcna3* expression was higher at P21 under all conditions. Overall, the results suggest that there are early developmental changes in microglia and their inflammatory responses that persist *in vitro*. However, if there are sex differences at these times, most were not manifest in isolated rat microglia.

## Materials and Methods

All procedures were performed in accordance with guidelines established by the Canadian Council on Animal Care; and were approved by the University Health Network Animal Care Committee (animal protocol #914).

### Microglia Cultures and Treatments

Sprague-Dawley rats (Charles River; St Constant, QC, Canada) were disaggregated by sex on the basis of the presence of gonads (P21) or anogenital distance (P1; (Jackson, 1912). For P1 animals, tail snips were sent to Transnetyx, Inc. (Cordova, TN, United States)^1^ where they confirmed the sex using real-time PCR with rat-specific primers for the *Sry* gene: GGGACAACAACCTACACACTATCAT (forward primer), TGTCCACAGGCTGTAAATAAATGCT (reverse primer).

#### Cell Cultures

Neonatal microglia (P1) were isolated using the same procedures as in our recent papers (Lam et al., 2017; Lively and Schlichter, 2018). In our hands, these methods yield 98–100% microglial purity, as determined by labeling with tomato lectin or antibodies against Iba1 or CD11b (e.g., Siddiqui et al., 2012, 2016; Lively et al., 2018). For instance, we very recently stained for CD11b and DAPI, and showed essentially pure microglia in these cultures (Lively and Schlichter, 2018).

Prepubertal (P21) microglia were isolated after rats were deeply anesthetized using isoflurane and killed by decapitation. About 400 mg of brain tissue from one cortical hemisphere was dissociated using a Neural Tissue Dissociation Kit (Miltenyi #130-092-628). Myelin was removed with myelin-specific magnetic beads (Miltenyi #130-105-634), and the cell suspension was incubated with CD11b magnetic microbeads (Miltenyi #130-105-634) to capture microglia. Microglia were seeded in MEM with 10% FBS and incubated for 2–3 days, at which time most were unipolar.

#### Stimulation

We chose the cytokines, their concentrations and duration of treatment based on numerous studies from our laboratory (e.g., Lam et al., 2017; Lively and Schlichter, 2018) and others (Spanaus et al., 1998). The rationale for using I+T is detailed in our recent paper (Lively and Schlichter, 2018). In brief, both cytokines are rapidly elevated in numerous *in vivo* rodent models of CNS damage and disease (Benveniste and Benos, 1995; Barcia et al., 2011; Woodcock and Morganti-Kossmann, 2013). Moreover, our recent *in vitro* studies show that I+T evokes numerous transcriptional and functional changes that could have important consequences for microglial roles after CNS damage (Siddiqui et al., 2016). IL-4 is well known to exert anti-inflammatory actions on rodent microglia (Colton, 2009) and there is considerable information about the molecular and functional changes it evokes. For instance, the dose of IL-4 used here increases migration, invasion (Lively and Schlichter, 2013), and Kv1.3 channel expression and current (Lam et al., 2017).

In the present study, microglia were left untreated (control, CTL) or incubated with 20 ng/mL IFNγ plus 50 ng/mL TNFα (I+T) or with 20 ng/mL IL-4. Treatments were 24 h for mRNA, protein and functional analyses (nitric oxide [NO] production, migration), as before (Lam et al., 2017; Lively and Schlichter, 2018). A longer time (30 h) was used for patch-clamp analysis to allow for channel trafficking and potential post-translational modifications, as before (Lam et al., 2017; Lively et al., 2018).

#### Staining

Procedures were the same as in our recent papers (Lam et al., 2017; Lively and Schlichter, 2018). In brief, microglia on coverslips (8 × 10^4^ cells/coverslip; 5–6 independent cultures used for each sex and age) were quickly washed in PBS, fixed in 4% paraformaldehyde (PFA; Electron Microscopy Sciences, Hatfield, PA, United States; Cat# 15710), washed again in PBS, and then permeabilized with 0.2% Triton X-100. The F-actin label, Acti-stain 488 phalloidin (1:100 in PBS; Cytoskeleton Inc., RRID:SCR_013532; Cat# PHDG1-A) was added for 1 h, and then the nuclear dye, 4′,6-diamidino-2-phenylindole (DAPI; 1:3000 in PBS; Sigma-Aldrich; Cat# D9542) was added for 5 min. Coverslips were mounted on glass slides with DAKO mounting medium (Agilent-Dako, RRID:SCR_013530; Cat# S302380-2) and stored in the dark at 4°C. Images were acquired with a Zeiss 880 confocal microscope (model LSM880; Zeiss, Oberkochen, Germany) and Zen software (version 2.3 SPI; Zeiss, Toronto, ON, Canada).

### Functional Assessment

#### Nitric Oxide Production

The colorimetric Griess assay was used to measure nitrite (Lam et al., 2017; Lively and Schlichter, 2018). Briefly, microglia (8 × 10^4^ cells/coverslip; 17–19 independent cultures for each sex at P1; 9–13 independent cultures for each sex at P21) were incubated for 24 h with I+T or IL-4 (as above) and then aliquots of the supernatants were added to 96-well plates containing 1% sulfanilic acid (Sigma-Aldrich; Cat#86090). After adding 0.1% *N*-(1-naphthyl)ethylene diamine dihydrochloride (Sigma-Aldrich; Cat#222488), the plates were incubated for 30 min in the dark. The resulting color change (absorbance at 570 nm) was quantified using a plate counter (Victor^3^ 1420, Perkin Elmer, Woodbridge, ON, Canada), and the nitrite concentration calculated by interpolation from a standard curve.

#### Migration

Microglia were seeded at 3 × 10^4^ cells/filter in 500 μL MEM with 2% FBS onto Transwell inserts bearing 8 μm-diameter holes (VWR; Cat# CA 62406-198), as before (Lively and Schlichter, 2013; Lam et al., 2017; Lively and Schlichter, 2018). Independent cultures for each sex numbered 24 at P1 and 7–11 at P21. After the cells were incubated for 30 min (37°C, 5% CO_2_), 500 μL solution (MEM with 2% FBS) was added to the lower wells, and a stimulating cytokine was added (I+T or IL-4, as above). 24 h later, the cells were fixed in 4% PFA (10 min) and rinsed 3× with PBS. Cells that had not migrated were removed by swirling the upper face of the insert with a Q-tip. The migrated cells were stained with 0.3% crystal violet (1 min), viewed at 20× magnification with an Olympus CK2 inverted microscope (Olympus, Tokyo, Japan) and summed from 5 random fields/filter. Then, the counts were normalized to the unstimulated (control) group.

### Transcription Analysis

Microglia (0.5–1 × 10^6^cells/coverslip; 5–7 independent cell cultures for each sex and age) were plated in 12-well culture plates and left untreated or stimulated for 24 h with 20 ng/mL IFNγ + 50 ng/mL TNFα or 20 ng/mL IL-4, as above. The remaining methods were as before (Lam et al., 2017). In brief, total RNA was extracted (TRIzol reagent; ThermoFisher Scientific; Cat# 15596018) and RNeasy Mini Kits (QIAGEN, Mississauga, ON, Canada; Cat# 74104). The gene expression assay (NanoString nCounter^TM^ technologies) was conducted at the Princess Margaret Genomics Centre (Toronto, ON, Canada)^2^ using 100 ng of extracted RNA from each sample. The code set, which was designed by NanoString, consists of capture and reporter probes (Supplementary Table 1). Raw data were analyzed (nSolver^TM^ Analysis Software ver3.0; RRID:SCR_00342), the background was subtracted using negative reporter probes, and irrelevant control genes were added to assess hybridization efficiency, detection range, and to calculate a scaling factor that was applied to all mRNA counts in each sample. A reference gene scaling factor was calculated using 5 housekeeping genes: Gapdh (glyceraldehyde 3-phosphate dehydrogenase), Gusb (glucuronidase beta), Hprt1 (hypoxanthine phosphoribosyltransferase 1), Rpl32 (ribosomal protein L32), and Sdha (succinate dehydrogenase complex flavoprotein subunit A. For statistical analysis, the normalized data were log2-transformed. In the Figures, transcript expression data are shown as normalized mRNA counts/100 ng of total RNA. In Supplementary Tables, control data are also shown as normalized mRNA counts, and then treatment effects (I+T; IL-4) are highlighted by showing fold changes.

### Western Blots

The methods for conducting and analyzing Western blots were essentially as before (Lam et al., 2017). Microglia were seeded (1–3 × 10^6^ cells/well; 5–12 independent cell cultures for each sex and age), and then treated with I+T or IL-4, as above. Cells were harvested, lysed in ice-cold RIPA and a mammalian protease inhibitor cocktail (Sigma-Aldrich; Cat# P3840), and centrifuged to remove insoluble material. Protein concentrations were determined with a Pierce^TM^ BCA protein assay (ThermoFisher Scientific; Cat# 23225), and then proteins were denatured (100°C for 5 min in a dry-bath incubator) in NuPage LDS sample buffer (ThermoFisher Scientific; Cat# NP0007) containing 5% 2-β-mercaptoethanol. 8% acrylamide gels were loaded with 10 μg protein/lane, which were separated by SDS-PAGE and transferred to a PVDF membrane, and then blocked for 2–3 h in 5% non-fat dry milk in Tris-Tween buffered saline (TTBS).

Protein levels were measured for exemplary pro- (iNOS, PYK2, COX-2) and anti-inflammatory (CD206) markers, as before (Lively and Schlichter, 2018). Primary antibodies were diluted in TTBS with 1% BSA and applied overnight at 4°C. They were: mouse anti-iNOS (1:250; Abcam Cat# ab49999, RRID:AB_881438), rabbit anti-PYK2 (1:500; Abcam Cat# ab32571, RRID:AB_777566), rabbit anti-COX-2 (1:1000; Abcam Cat# ab15191, RRID:AB_2085144), and rabbit anti-CD206 (1:2000; Abcam, Cat# ab64693, RRID:AB_1523910). Horseradish peroxidase-labeled secondary antibodies in 1% BSA-TTBS were applied for 1 h (1:3000; Cedarlane, Burlington, ON, Canada, RRID:SCR_004462; anti-rabbit IgG: Cat# CLCC42007; anti-mouse IgG: Cat # CLCC30207), and after repeated washing, the membranes were treated with GE Healthcare ECL Start Western Blotting Detection Reagent (Sigma-Aldrich; Cat# GERPN3243). Protein band intensities were determined with a ChemiDoc XRS System (Bio-Rad).

Total protein normalization was used to compare changes using the Coomassie blue staining method (Welinder and Ekblad, 2011). We previously found that this method was preferable to a single reference protein (e.g., β actin) because such ‘housekeeping’ proteins can change with microglial activation states (Lam et al., 2017). A 0.1% solution of Coomassie Brilliant Blue G (Sigma-Aldrich; Cat# B8522) was applied (1 min), and then slides were de-stained (2 min) in acetic acid/methanol/water (1:5:4) and air-dried. Blots were imaged with a ChemiDocTM XRS System, and then analyzed using Image Lab (ver.5.2.1; RRID:SCR_014210) to identify gel lanes and bands of interest, and to subtract the background and determine signal intensities of identified bands. For each blot, the band of interest was normalized to total Coomassie-blue stained protein in that lane (as a loading control), and then fold-changes were calculated with respect to unstimulated (control) microglia. Uncropped images of representative blots are shown in Supplementary Figure 1.

### Patch-Clamp Electrophysiology

Experiments were conducted on prepubertal microglia isolated at P21. Immediately after isolation, microglia were plated on glass coverslips (∼7 × 10^4^ cells/coverslip) and then incubated in tissue culture medium, as above. Recording methods were as before (Lam et al., 2017; Lively et al., 2018). Coverslips were placed in a 300 μL perfusion chamber (Model RC-25, Warner Instruments, Hamden, CT, United States) containing bath solution (in mM): 125 NaCl, 5 KCl, 1 CaCl_2_, 1 MgCl_2_, 10 HEPES, 5 D-glucose, adjusted to pH 7.4 and 290–300 mOsm. Whole-cell K^+^ currents were recorded at room temperature using fire-polished patch pipettes (4–8 MΩ resistance) and a pipette solution consisting of (in mM): 40 Cl, 100 KAsp, 1 MgCl_2_,10 HEPES, 2 MgATP; pH 7.2, 290–300 mOsm. Data were acquired with an Axopatch 200A amplifier and DigiDATA 1322A board (Molecular Devices, Sunnyvale, CA, United States); filtered at 5 Hz and analyzed using pCLAMP (ver10; RRID:SCR_011323). Series resistance and capacitance transients were compensated on-line, junction potentials were calculated with a utility in pCLAMP (and corrected in the figures), and the data were analyzed using Origin (ver 9.0; RRID:SCR_014212; Microcal, Northampton, MA, United States) and GraphPad Prism (ver6.0; RRID:SCR_002798; La Jolla, CA, United States).

### Statistics

Data are expressed as mean ± SEM in bar graphs and mean ± SD in scatterplots and tables for the number of biological replicates indicated. Statistical analyses were conducted in GraphPad Prism. To identify expression changes induced by stimulation in either a sex- or age-dependent manner, log2-transformed counts obtained from NanoString (described above) were analyzed by two-way ANOVA with Fisher’s LSD test. The resulting *p*-value for each gene was then corrected for multiple comparisons using a 5% false discovery rate (FDR; Benjamini and Yekutieli, 2001) in the program R (version 3.3.1; RRID:SCR_001905). For all other data (i.e., NO production, migration, Western blotting and patch-clamp electrophysiology), data were analyzed by a two-way ANOVA followed by Tukey’s *post hoc* test. Differences were considered significant if *p* < 0.05.

## Results

### Neonatal and Prepubertal Female and Male Microglia Respond to IFNγ + TNFα and IL-4

**FIGURE 1:**
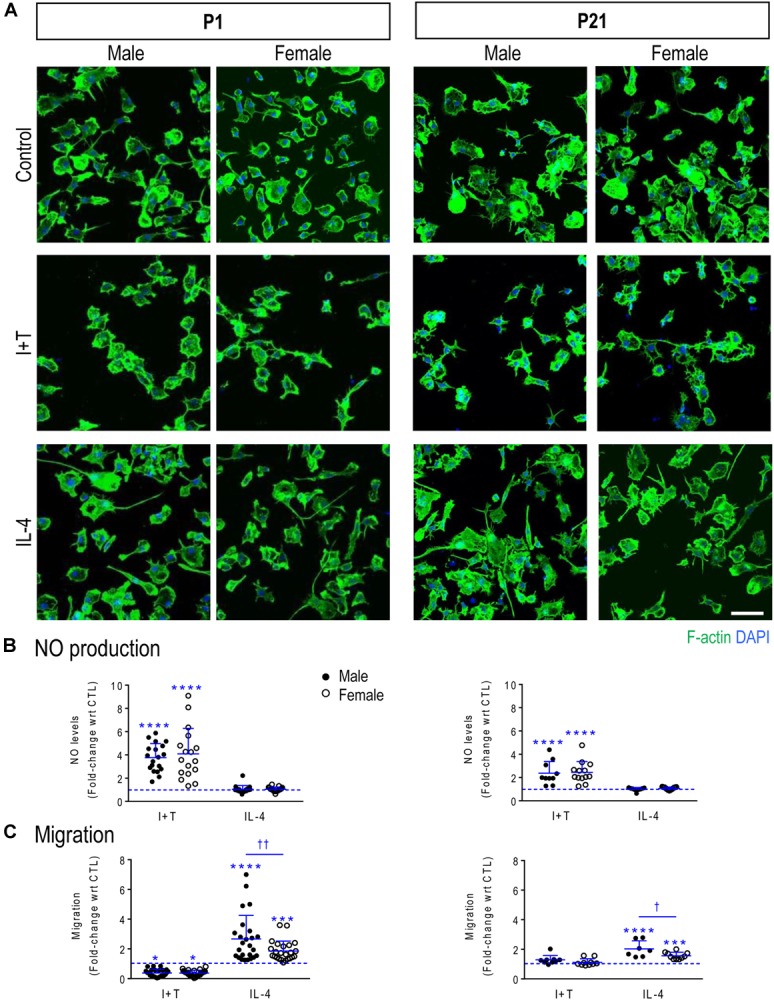
Verifying that primary rat microglia responded to IFNγ + TNFα and to IL-4 at P1 and P21. Male and female neonatal (P1) and prepubertal (P21) microglia were sex-segregated (see Materials and Methods). **(A)** Representative fluorescence images of primary rat microglia (unstimulated, CTL) and 24 h after treatment with I+T or IL-4. Microglia were fixed and stained for F actin (phalloidin; green) and nuclei (DAPI; blue). Scale bar, 50 μm. **(B)** Cumulative NO production for the 24 h period after stimulation. Each circle represents an individual culture (closed, male; open, female). **(C)** The activation state differently affects migration of microglia at P1 and P21. All graphical results are expressed as fold changes normalized to unstimulated cells, which are indicated by dashed lines. Individual values are plotted, mean ± SD indicated, and differences shown are with respect to control microglia (^∗^) and between sexes (†). One symbol of either type indicates *p* < 0.05; two symbols, *p* < 0.01; three symbols, *p* < 0.001; four symbols, *p* < 0.0001.

Microglia morphology was examined as an indicator of their general health and responsiveness to cytokines (Figure 1A). While staining fixed cells only shows a snapshot in time, unstimulated cells were predominantly unipolar with an F-actin-rich lamellum and a uropod, and occasionally more rounded with spiky processes. This is similar to our studies showing that unipolar microglia were highly migratory (e.g., Siddiqui et al., 2012; Lively and Schlichter, 2013, 2018; Siddiqui et al., 2014; Lam et al., 2017). Importantly, cells did not display apoptotic blebs, and the nuclear morphology was not pyknotic under any condition (age, sex, treatments). We did not conduct a detailed morphological analysis because they are highly malleable (live imaging); and we already know that their shape need not correlate with gene expression patterns. For instance, unstimulated, IL-4- and IL-10-treated rat microglia are morphologically similar but differ greatly in gene expression (Siddiqui et al., 2016; Lam et al., 2017). We then noted I+T-evoked shape changes simply as a means of verifying that microglia from both sexes and ages had responded, and observed that most rounded up and formed chain-like groupings. IL-4 did not greatly affect their shape. While the cell bodies might appear larger, visual inspection is not a reliable measure of surface area because retraction of processes, membrane ruffling and changes in cell height can occur without changing membrane area. As described in the section on K^+^ currents (below), cell capacitance is a better indicator of size changes; capacitance did not change after IL-4 (Lam et al., 2017). Prepubertal (P21) microglia had variable shapes, ranging from unipolar to flat and angular, and they responded to the cytokines in a similar manner to neonatal cells. Thus, instead of relying on morphology, we quantified two known correlates of their activation state, nitric oxide (NO) production and migratory capacity.

Increased NO production (due to up-regulation of *Nos2* mRNA and iNOS protein) is commonly used to indicate a pro-inflammatory microglial activation state. Initial evidence that unstimulated microglia were not ‘activated’ is that NO production (monitored as nitrite accumulation in the culture medium) was low and similar regardless of sex or age. Nitrite concentrations in the medium were 2.00 μM ± 0.41 (SD; *n* = 19) for P1 males; 2.15 ± 0.42 (*n* = 17) for P1 females; 2.31 ± 0.43 (*n* = 10) for P21 males; and 2.33 ± 0.49 (*n* = 13) for P21 females. There were no statistical differences (two-way ANOVA with Tukey’s test). These data are also consistent with our earlier studies showing low NO production by combined-sex neonatal microglia (e.g., Sivagnanam et al., 2010; Lam et al., 2017; Lively and Schlichter, 2018). Further evidence that unstimulated microglia were not activated is presented in the sections on transcriptional responses, below.

A 24-h treatment with I+T increased NO production in both sexes and at both ages. For P1 cells, I+T evoked about a 4-fold increase in NO production: 3.8 ± 1.2 fold in males (*n* = 19 individual cultures) and 4.1 ± 2.2 fold in females (*n* = 17) (Figure 1B). At P21, the increase was somewhat smaller: 2.4 ± 1.0 fold in both sexes (*n* = 10–13). As expected, IL-4 had no effect on NO production. These results extend our recent studies using neonatal rat microglia of combined sexes (Lam et al., 2017; Lively and Schlichter, 2018). For unstimulated P1 microglia, migration was 50% higher in males (30 ± 23 cells/5 fields of view; mean ± SD, *n* = 19) than in females (20 ± 9, *n* = 17) but the variability was too large in males to confirm a statistically significant difference. There were some sex and age differences. At P1, migration was decreased by I+T and increased by IL-4 in both sexes but male IL-4-treated cells migrated 44% more than female cells (Figure 1C). For unstimulated P21 cells, migration was comparable in males (41 ± 27 cells/5-fields of view, *n* = 7) and females (47 ± 21, *n* = 10) but I+T did not reduce migration. At P21, the sex dimorphism was preserved, with IL-4 increasing migration of male cells 29% more than females.

### Sex Comparison of Transcriptional Responses

We routinely use targeted analysis of >50 genes to assess responses of neonatal microglia from rats and mice to pro-inflammatory (lipopolysaccharide [LPS], I+T) and anti-inflammatory stimuli (IL-4, IL-10, TGFβ1) (e.g., Lam et al., 2017; Lively and Schlichter, 2018; Lively et al., 2018). Our previous studies always combined microglia from both sexes. Here, we separated males and females to examine responses of 69 genes to I+T and IL-4. The starting state will affect the ability of microglia to respond to stimuli. Gene expression data are useful to define the initial state before inducing various perturbed or ‘activated’ states. As in our previous studies of mixed sex microglia (some cited above), unstimulated P1 rat microglia were in a relatively non-inflammatory state (which some would call ‘resting’), as judged by low expression of many inflammatory molecules in both sexes. For instance, they had low expression (arbitrary cutoff, <250 mRNA counts/100 ng RNA) of pro-inflammatory (*Casp1*, *Cd274*, *Cxcl10*, *Ifnb1*, *Ifng*, *Il1a*, *Il1b*, *Il6*, *Nos2*, *Ptgs2*, *Tnf*) and anti-inflammatory genes (*Arg1*, *Ccl22*, *Cd163*, *Il4*, *Il10*, *Pparg*, *Retnla*).

**FIGURE 2:**
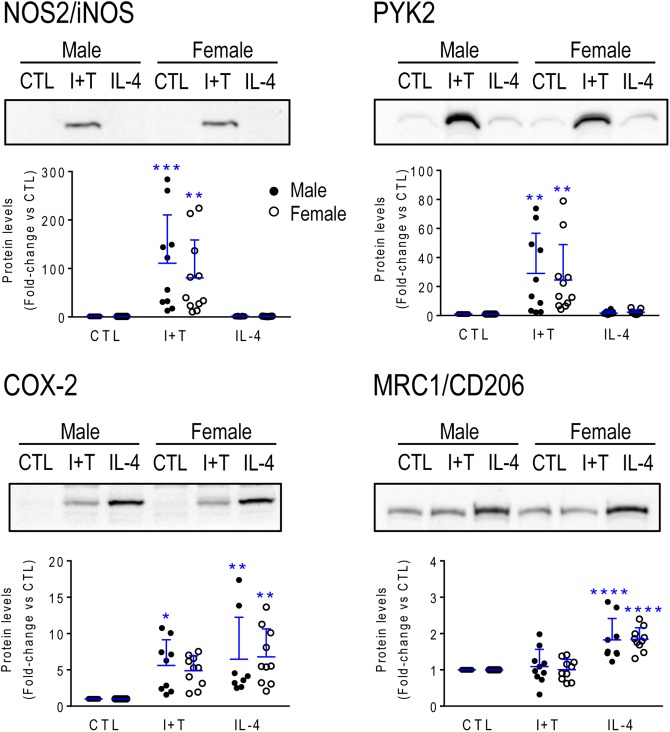
Neonatal (P1) microglia: Exemplary pro- and anti-inflammatory proteins. Rat microglia were harvested 24 h after treatment with IFNγ + TNFα (I+T) or IL-4. **(Upper)** Representative Western blots for the pro-inflammatory markers, iNOS, COX-2 and PYK2, and the anti-inflammatory marker, MRC1/CD206. **(Lower)** Individual values show fold-changes with respect to unstimulated (control) microglia and the mean ± SD is indicated. Differences from control microglia are shown as: ^∗^*p* < 0.05; ^∗∗^*p* < 0.01; ^∗∗∗^*p* < 0.001, and ^∗∗∗∗^*p* < 0.0001.

Next, sex-segregated microglia were stimulated for 24 h with I+T or IL-4 to ask whether their transcriptional responses differed. Many gene expression studies examine a single time point and we chose 24 h because it is commonly used to investigate changes in microglial gene expression (Kobayashi et al., 2013; Zhang et al., 2015) and it facilitates comparison with our recent studies of I+T and IL-4 at 24 h that also included pilot studies showing similar responses at 6 h (Lam et al., 2017; Lively and Schlichter, 2018). [Note: Because we saw no sex differences, the segregated gene expression data and statistical outcomes are in Supplementary Tables 2–5 for P1 and 6–9 for P21.] For P1 microglia, none of the genes showed sex differences in their responses to I+T or IL-4, and their responses were similar to our earlier combined-sex neonatal cultures. For instance, I+T increased genes associated with pro-inflammatory responses (*Casp1*, *Cybb*, *Kcna3*, *Kcnj2*, *Ncf1*, *Nos2*, *Ptgs2*, *Ptk2b*, *Tnf*) and decreased homeostatic molecules (*P2ry12*, *Cx3cr1*). IL-4 increased *Arg1*, *Ccl22* and *Mrc1*. Next, we examined changes at the protein level for four of these molecules (Figure 2). Consistent with the transcript changes, I+T increased NOS2, PYK2 and COX-2 (*p* value for female cells was 0.07), and IL-4 increased CD206. IL-4 also increased COX-2 protein, consistent with the increases in mRNA (8.4-fold in males, 11.3-fold in females), but protein changes did not reach statistical significance. No sex differences were evident.

The present study included sex-comparisons of 19 genes we had not previously examined; i.e., pro-inflammatory (*Cd274* /PD-L1, *Cxcl10*, *Ifnb1*, *Il1a*) and anti-inflammatory molecules (*Il13*, *Il13ra1*, *Pprc1/*PRC), members of the inflammasome (*Nlrp3*, *Pycard*), immune modulators and genes related to microglial physiology (*Cd200r1*, *Csf1*, *F2r*/PAR-1, *Hmox1*/Heme oxygenase 1, *Lcn2/*NGAL, *Mmp9*, *Nfe2l2*/NRF2, *P2rx4*, *Pdcd1*/PD-1, *Tfrc)*. There were numerous responses to cytokines; e.g., at P1, I+T increased *Cd274*, *Cxcl10*, *Il1a*, *Il4r*, *Il13ra1*, *Nlrp3*, *Hmox1*, *Nfe2l2*, and *P2rx4*; and decreased *Cd200r1* and *Csf1*. IL-4 increased *Csf1* and *F2r*, decreased *Il13ra1* and *P2rx4*; and surprisingly, it also increased the pro-inflammatory genes, *Cd274* and *Il1a*. Again, there were no sex differences at either age; hence, the data are shown in Supplementary Tables 2–9.

### Age-Related Differences in Gene Expression and Responses

**FIGURE 3:**
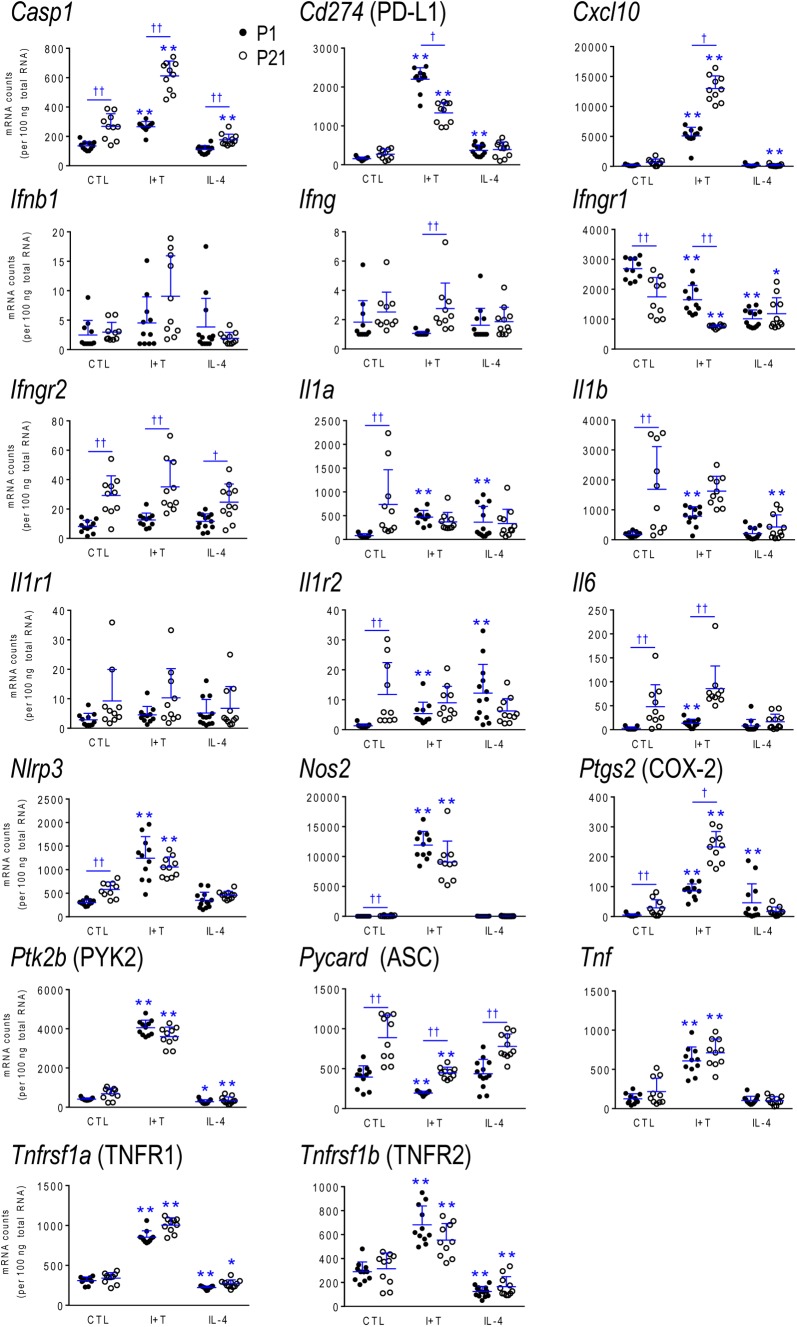
Age-dependent transcriptional responses: Pro-inflammatory mediators. Rat microglia were unstimulated (CTL) or stimulated for 24 h with IFNγ and TNFα (I+T) or IL-4. Transcript expression of each gene is shown as mRNA counts normalized to two housekeeping genes (see Materials and Methods). For clarity, protein names are included for some genes. The scatterplots show individual values and the mean ± SD. Differences shown are with respect to control microglia (^∗^) and between P1 and P21 cells (†). One symbol of either type indicates *p* < 0.05; two symbols, *p* < 0.01.

**FIGURE 4:**
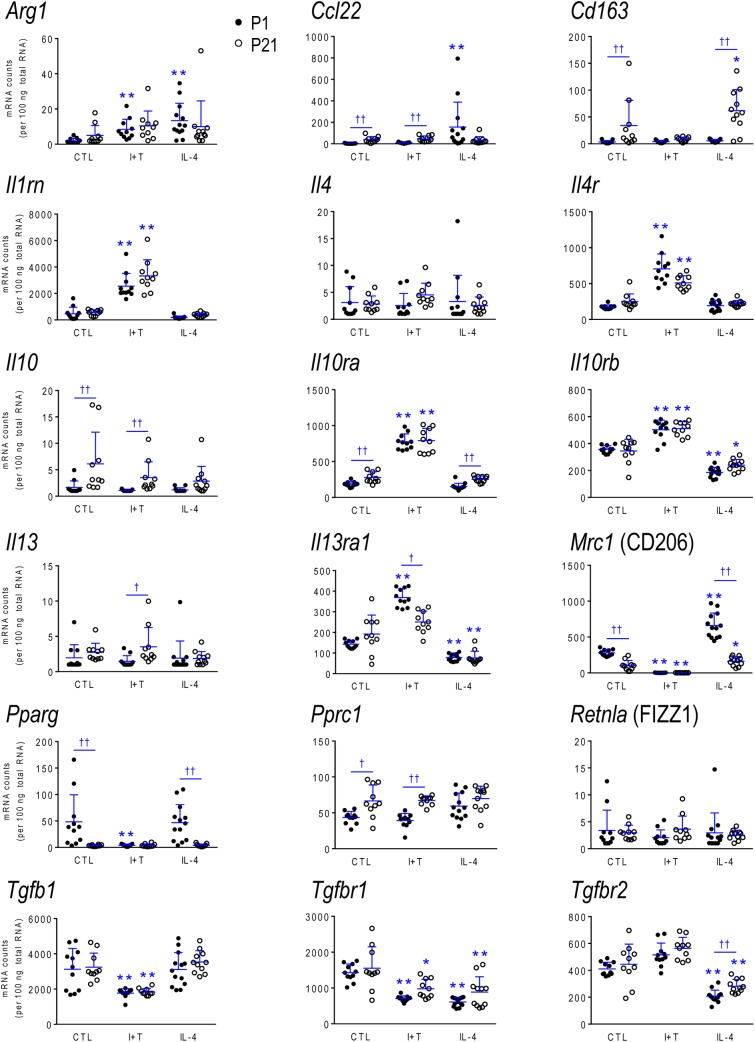
Age-dependent transcriptional responses: Anti-inflammatory mediators. As in Figure 2, microglia were stimulated for 24 h with IFNγ and TNFα (I+T) or IL-4. The scatterplots show individual mRNA values (normalized; see Materials and Methods) and the mean ± SD. Differences shown are with respect to control microglia (^∗^) and between P1 and P21 cells (†). One symbol of either type indicates *p* < 0.05; two symbols, *p* < 0.01.

**FIGURE 5:**
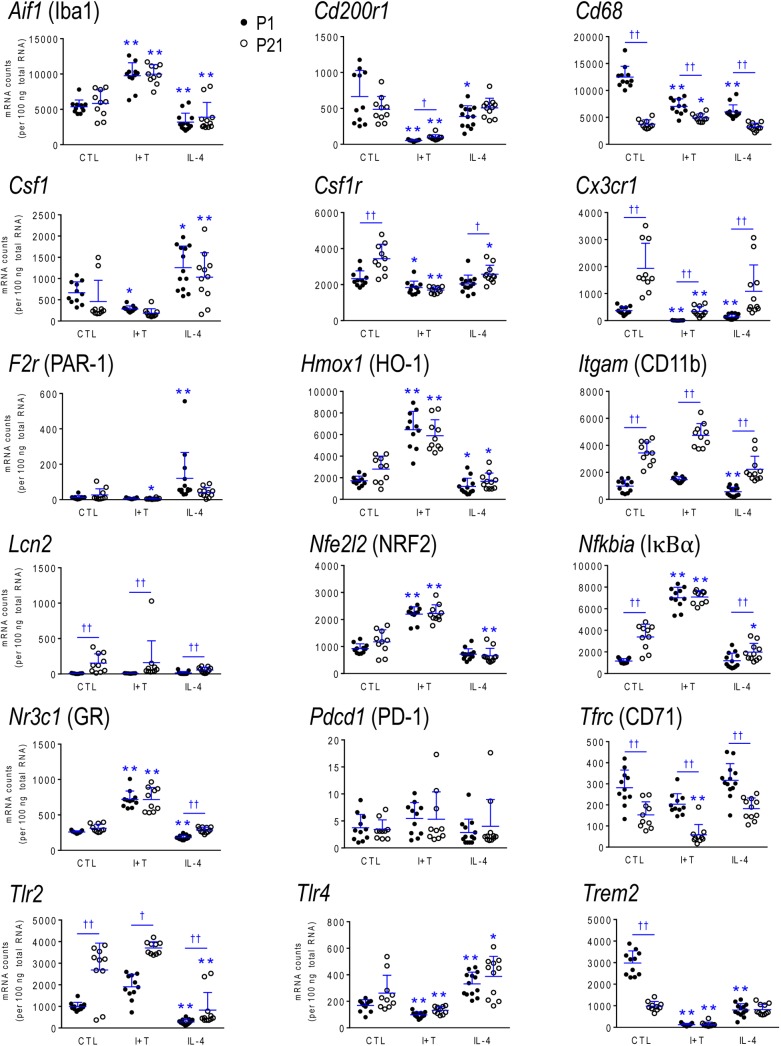
Age-dependent transcriptional responses: Microglia markers and modulators. As in Figure 2, microglia were stimulated for 24 h with IFNγ and TNFα (I+T) or IL-4. The scatterplots show individual mRNA values (normalized; see Materials and Methods) and the mean ± SD. Differences shown are with respect to control microglia (^∗^) and between P1 and P21 cells (†). One symbol of either type indicates *p* < 0.05; two symbols, *p* < 0.01.

**FIGURE 6:**
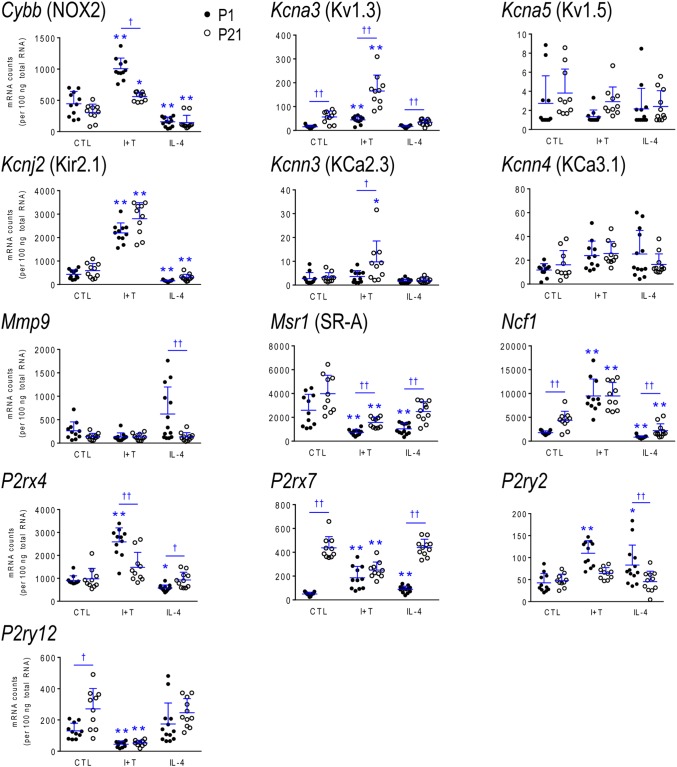
Age-dependent transcriptional responses: Molecules related to selected physiological functions. As in Figure 2, microglia were stimulated for 24 h with IFNγ and TNFα (I+T) or IL-4. The scatterplots show individual mRNA values (normalized; see Materials and Methods) and the mean ± SD. Differences shown are with respect to control microglia (^∗^) and between P1 and P21 cells (†). One symbol of either type indicates *p* < 0.05; two symbols, *p* < 0.01.

**FIGURE 7:**
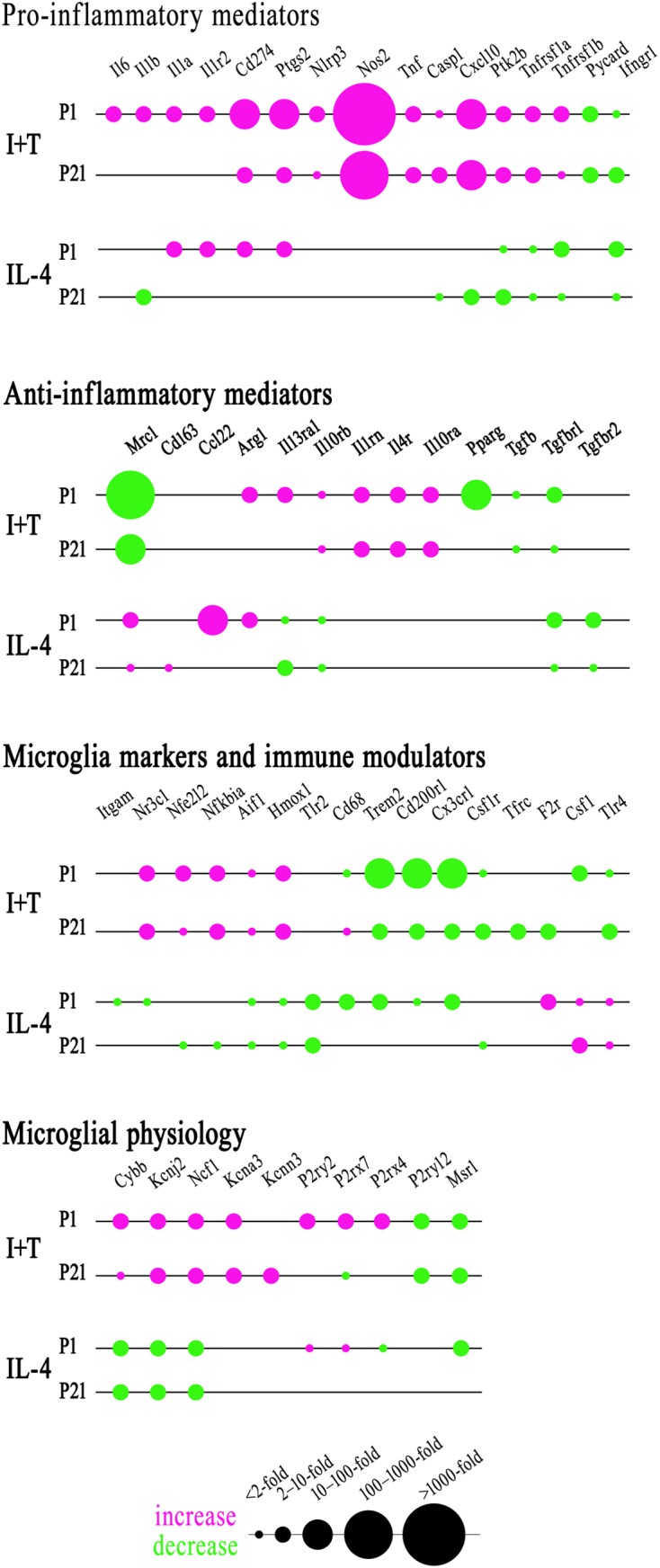
Summary of the magnitude and direction of gene expression changes in neonatal (P1) and prepubertal (P21) microglia. Sexes were combined because no sex differences were identified at either age. To create the bubble chart, fold changes were sorted into bins according to the ranges stated on the figure. Only genes showing changes in expression were included. See Figures 3–6 for full data and significance levels. The chart shows fold changes evoked by I+T or IL-4 relative to control values (magenta = increases; green = decreases).

As there were no sex differences, to assess potential age-related differences (P1 *versus* P21), we then combined data from male and female microglia. Numerical data and statistics are shown in Figures 3–6, and organized into the same four categories: pro-inflammatory (Figure 3), anti-inflammatory (Figure 4), microglial markers and immune modulators (Figure 5), and genes related to microglia physiological functions (Figure 6). Then, to highlight similarities and differences, responses to I+T and IL-4 are pictorially summarized according to the age (Figure 7). For completeness, age comparisons of the sex-segregated data and accompanying statistics are shown in Supplementary Tables 10–17.

#### Baseline Levels in Unstimulated Microglia

Because microglia were isolated by different methods at the two ages, it is important to compare their starting state as well as responses to I+T and IL-4. P1 and P21 microglia had comparable baseline levels for many genes from all four categories: pro-inflammatory (*Cd274, Cxcl10*, *Ifnb1*, *Ifng*, *Il1r1*, *Ptk2b*, *Tnf*, *Tnfrsf1a*, *Tnfrsf1b*), anti-inflammatory (*Arg1*, *Il1rn*, *Il4*, *Il4r*, *Il10rb*, *Il13*, *Il13ra1*, *Retnla*, *Tgfb1*, *Tgfbr1*, *Tgfbr2*), markers and immune modulators (*Aif1*, *Cd200r1*, *Csf1*, *F2r*, *Hmox1*, *Nfe2l2*, *Nr3c1*, *Pdcd1*, *Tlr4*), and genes related to microglial physiology (*Cybb*, *Kcna5*, *Kcnj2*, *Kcnn3*, *Kcnn4*, *Mmp9*, *Msr1, P2rx4*, *P2ry2*). However, other genes differed and had higher basal expression at P21: pro-inflammatory (*Casp1*, *Ifngr2*, *Il1a*, *Il1b*, *Il1r2*, *Il6*, *Nlrp3*, *Nos2*, *Ptgs2*, *Pycard*), anti-inflammatory (*Ccl22*, *Cd163*, *Il10*, *Il10ra*, *Pprc1*), markers and modulators (*Csf1r*, *Cx3cr1*, *Itgam*, *Lcn2*, *Nfkbia*, *Tlr2*), and genes related to microglial physiology (*Kcna3*, *Ncf1*, *P2rx7*, *P2ry12*). A few genes had lower baseline expression at P21 (*Ifngr1*, *Tfrc*, *Mrc1*, *Pparg*, *Cd68*, *Trem2*). When all genes are considered, it is evident that P21 cells are not simply more or less ‘activated’ [Eleven genes were expressed at low levels (< 50 mRNA counts/100 ng RNA) at both ages and were not altered by I+T or IL-4 (*Ifnb1*, *Ifng*, *Ifngr2*, *Il1r1*, *Il4*, *Il10*, *Il13*, *Kcna5*, *Kcnn4*, *Retnla*, and *Pdcd1*) and will not be discussed further].

#### I+T Treated Microglia

Many genes showed similar general responses, or lack thereof, at P1 and P21, with similarities in 12/16 pro-inflammatory genes (Figures 3, 7), 7/13 anti-inflammatory genes (Figures 4, 7), 11/16 microglial markers and modulators (Figures 5, 7), and 6/10 genes related to microglial physiology (Figures 6, 7). After omitting the non-responding genes, 30% of the I+T-affected genes showed nearly identical transcript levels at P1 and P21 both before and after stimulation. Transcripts that increased were *Aif1*, *Hmox1*, *Il1rn*, *Il4r*, *Il10rb*, *Kcnj2*, *Nfe2l2*, *Nr3c1*, *Ptk2b*, *Tnf*, *Tnfrsf1a*, *Tnfrs1b*. Decreases were seen in *Tgfb1*, *Tgfbr1* and *Tlr4*.

Some age-dependent differences in responses to I+T also emerged, with four patterns seen. (i) Those in which baseline levels differed with age but became similar after I+T treatment. Transcript levels increased for *Il10ra*, *Ncf1*, *Nfkbia*, *Nlrp3* and *Nos2*; and decreased for *Csf1r*, *Mrc1*, *P2ry12*, and *Trem2*. Note that baseline levels of most of these molecules were higher at P21 (except *Mrc1* and *Trem2* were higher at P1). (ii) Some genes showed the same direction of response to I+T but age-related differences in the basal levels resulted in differences after I+T. Again, expression was often higher at P21. Expression increased for *Casp1*, *Kcna3* and *Ptgs2*; decreased for *Cx3cr1*, *Ifngr1* and *Pycard*; and was unchanged for *Ccl22*, *Cd11b*, *Lcn2*, *Pprc1* and *Tlr2*. (iii) For a few genes, unstimulated levels were comparable at P1 and P21, but responses to I+T were greater at one age. Greater P1 responses were the increases in *Cd274* and *Cybb* and decreases in *Cd200r1* and *Msr1*. The only larger P21 response was the increase in *Cxcl10*. (iv) Some responses were specific to one age. Only at P1 did I+T increase *Arg1*, *Il13ra1*, *P2rx4* and *P2ry2*, and decrease *Csf1*. Only at P21 did I+T increase *Kcnn3* and decrease *F2r* and *Tfrc*. At P1, *Il1a*, *Il1b*, *Il1r2* and *Il6* were selectively increased; but interestingly, P21 microglia already had higher baseline levels and were not increased by I+T. At P1, I+T decreased *Pparg* but baseline levels at P21 were already lower. Two opposite I+T-mediated responses were seen between the two ages. *Cd68*, a lysosomal marker often used as an indicator of phagocytosis *in vivo* (Damoiseaux et al., 1994), was reduced by I+T at P1 (from a much higher baseline level) but increased at P21. The *P2rx7* transcript level increased at P1, but decreased at P21 (from a much higher baseline level).

#### IL-4 Treated Microglia

Some responses were similar at both ages; 4/16 pro-inflammatory genes (Figures 3, 7), 5/13 anti-inflammatory genes (Figures 4, 7), 5/16 microglia markers and immune modulators (Figures 5, 7) and 3/10 genes related to microglial physiology (Figures 6, 7). However, numerous similarities (14 genes) were simply a lack of response to IL-4.

For the IL-4-responding genes, several patterns were seen. (i) 29% (12/41) of genes showed comparable transcript levels at both ages before and after stimulation with IL-4. Unlike I+T, the IL-4-induced changes were mainly decreases; i.e., in *Aif1*, *Cybb*, *Hmox1*, *Il10rb*, *Il13ra1*, *Kcnj2*, *Ptk2b*, *Tgfbr1*, *Tnfrsf1a* and *Tnfrsf1b*, although *Csf1*, and *Tlr4* increased. (ii) There were some age-related differences that suggest developmental gene regulation. While the direction of the IL-4 response was the same, some transcript levels differed before and/or after adding IL-4; i.e., the amount of increase in *Mrc1* and decreases in *Ifngr1*, *Ncf1*, *Tgfbr2*, and *Tlr2*. (iii) Responses at P1 only. IL-4 increased some anti-inflammatory markers (*Arg1*, *Ccl22*) and purinergic receptors (*P2rx7*, *P2ry2*) but surprisingly, it also increased several pro-inflammatory molecules (*Cd274*, *F2r*, *Il1a*, *Il1r2*, *Ptgs2*). Also surprising was that IL-4 decreased molecules related to microglial quiescence (*Cd200r1*, *Cx3cr1*) and phagocytosis (*Cd11b*, *Cd68*, *Msr1*, *Nr3c1*, *P2rx4*, *Trem2*). [*Cd68* and *Trem2* were reduced from higher baseline levels at P1.] (iv) Responses at P21 only. IL-4 decreased expression of some pro-inflammatory genes (*Casp1*, *Cxcl10*, *Il1b*) and immune modulators (*Csf1r*, *Nfe2l2*, *Nfkbia*), of which four (*Casp1*, *Csf1r*, *Il1b*, *Nfkbia*) had higher baseline levels at P21. *Cd163* was the only IL-4-mediated increase specific to prepubertal microglia. Overall, at both ages, IL-4 decreased expression of several genes involved in general activation (*Aif1*), pro-inflammatory responses (*Kcnj2, Ptk2b*) and oxidative stress (*Cybb*, *Hmox1*). IL-4 also decreased both pro- and anti-inflammatory receptors (*Il10rb*, *Il13ra1*, *Tgfbr1*, *Tnfrsf1a*, *Tnfrsf1b*) suggesting that it skews microglia toward a refractory state.

**FIGURE 8:**
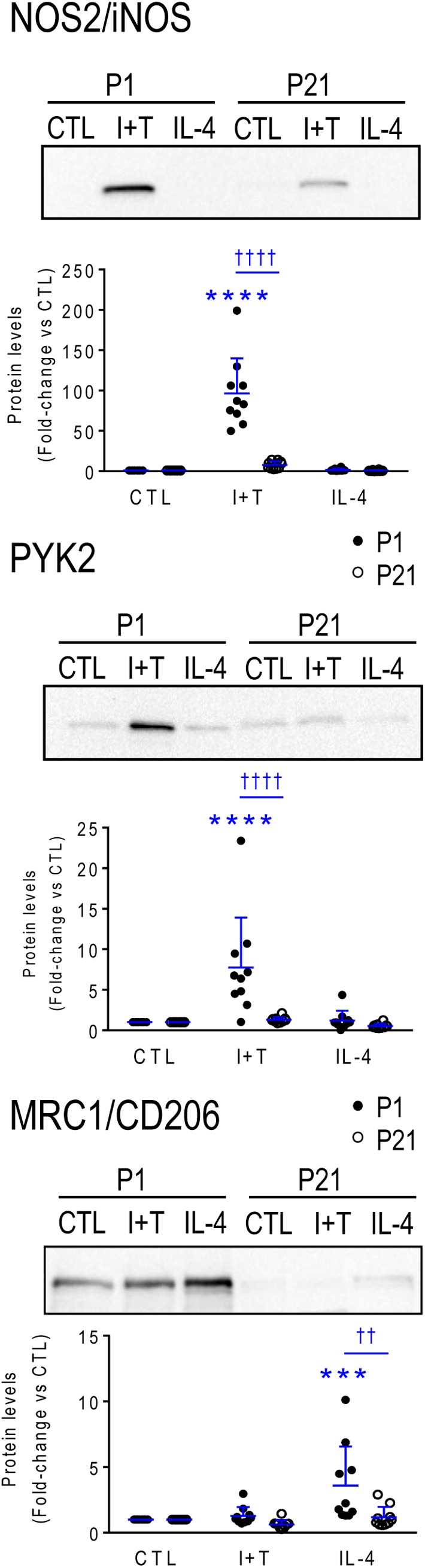
Neonatal (P1) versus prepubertal (P21) microglia: Exemplary pro- and anti-inflammatory proteins. Rat microglia were harvested 24 h after treatment with IFNγ + TNFα (I+T) or IL-4. **(Upper)** Representative Western blots for the pro-inflammatory markers, iNOS and PYK2, and the anti-inflammatory marker, MRC1/CD206. **(Lower)** Individual values of fold-changes with respect to unstimulated (control) microglia and the mean ± SD are shown. Significant differences are shown between control and treated cells (^∗^) and between P1 and P21 microglia (†). Two symbols of either type indicate *p* < 0.01; 3 indicates *p* < 0.001 and 4 indicates *p* < 0.0001.

Again, age-related responses of a few hallmark proteins were compared (Figure 8). As expected from the higher *Nos2* mRNA and NO production in P1 microglia after I+T stimulation, P1 microglia had higher levels of NOS2 protein. Also expected in P1 microglia, were the larger I+T-mediated increase in PYK2 protein, and larger IL-4-mediated increase in MRC1.

### Kv and Kir Currents in Prepubertal (P21) Microglia

Rat microglia express outward-rectifying Kv and inward-rectifying Kir currents, which have been extensively characterized in combined-sex neonatal microglia (see Discussion). Before undertaking the present study on prepubertal (P21) microglia, we conducted a pilot study on unstimulated P1 rat cells. There was no sex difference in the total Kv current amplitude: at +40 mV it was 15.1 ± 5.0 pA/pF (mean ± SEM; *n* = 6) in females and 12.7 ± 1.7 pA/pF (*n* = 15) in males.

This was not surprising because, in our many studies on ion currents in neonatal rat microglia, we have observed relatively small amplitude variations without evidence of a bimodal distribution that would suggest sex differences. Those studies include Kv and Kir currents after I+T or IL-4 treatment of mixed-sex microglia (Lam and Schlichter, 2015; Lam et al., 2017; Lively et al., 2018). Furthermore, the only age difference in mRNA expression for Kv and Kir channels was that *Kcna3* (Kv1.3) expression was higher at P21. Therefore, we next addressed potential sex differences in Kv and Kir currents, with and without stimulation by I+T or IL-4.

#### Kv Current

**FIGURE 9:**
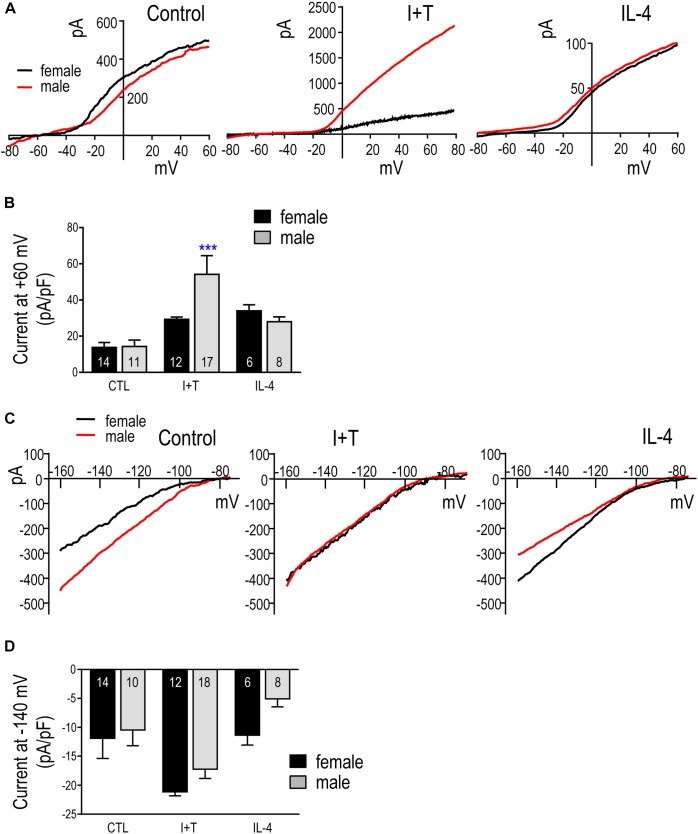
Kv and Kir currents in prepubertal (P21) microglia. **(A)** Representative whole-cell outward-rectifying Kv currents from male and female P21 microglia at 24 h in untreated cells (control) or after treatment with I+T or IL-4. For each recording, the voltage was ramped to +40 mV from a holding potential of –110 mV. **(B)** Summary of Kv current density (pA/pF) measured at +40 mV. Graphical summaries in **(B,D)** show mean ± SEM for the number of replicates indicated on each bar; and were analyzed by two-way ANOVA and Bonferroni *post hoc* test. ^∗∗∗^*p* < 0.001 indicates a difference from the control current. **(C)** Representative inward-rectifier (Kir) currents at 24 h in untreated microglia (control) or after treatment with I+T or IL-4. From a holding potential of –110 mV, the voltage was ramped from –160 to –60 mV. **(D)** Summary of peak Kir inward current density (pA/pF) measured at –140 mV.

Figure 9A shows representative Kv current traces from untreated (control) P21 male and female microglia in response to a voltage ramp, which gives a direct read-out of the current-versus-voltage (I–V) relationship. Control I–V curves were similar to our previous papers on unsexed rat microglia from neonates (see references above) and adults (Lively et al., 2018), and I+T increased the current in the male cell. IL-4 appeared to increase the current in microglia of both sexes. In choosing to assess the total Kv current, we recognize that more than one Kv channel type is likely involved (see Discussion). That is, only about half the current was blocked by the potent Kv1.3 blocker, agitoxin-2 (pilot study, not shown). While the remaining Kv current was not identified, we previously observed an additional Kv current (Lam et al., 2017) and also showed that Kv1.5 contributes to the microglia current in *ex vivo* tissue prints made from rat brain slices (Kotecha and Schlichter, 1999). Figure 9B shows a statistical comparison of male and female P21 microglia. The Kv current amplitude at +40 mV (in pA) was normalized to the cell size (membrane capacitance; in pF), as before (Lam et al., 2017; Lively et al., 2018). Capacitance is a useful way of accounting for cell size because it is independent of shape changes, such as retraction of cell processes, membrane ruffling, and loss of the lamellum that occurs after I+T treatment. In unstimulated (control) microglia, the Kv amplitude was the same in females and males. While there were apparent increases in Kv current in response to I+T and IL-4, the only statistically significant sex difference was that males had a nearly 2-fold higher response to I+T.

#### Kir Current

Figure 9C shows typical Kir currents in female and male P21 microglia. They are very similar to isolated Kir2.1 currents in neonatal (Lam and Schlichter, 2015; Lam et al., 2017) and adult rat microglia (Lam et al., 2017). For the analysis in Figure 9D, the Kir current was measured at –140 mV and normalized to cell size (capacitance, as above). While there were no statistically significant sex differences, there was a clear trend toward a larger response of female cells to IL-4.

#### TGFβ1 Response

Finally, although not part of this overall study, but for comparison with our recent paper on TGFβ1-treated rat and mouse microglia (Lively et al., 2018), we report effects of TGFβ1 treatment on the K+ currents in P21 rat microglia. Although the total Kv current at +40 mV was the same (888 ± 196 pA in males; 887 ± 171 in females), the cell size (capacitance) differed (32.9 ± 2.3 pF in males; 19.4 ± 1.5 pF in females) and, consequently, the size-adjusted current (pA/pF) was larger in females (46.4 ± 8.7 pA/pF; *n* = 14) than in males (29.5 ± 8.2 pA/pF; *n* = 9). For the Kir current, there was no sex difference after TGFβ1 treatment: it was 15.3 ± 3.7 pA/pF (*n* = 14) in females, and 11.8 ± 2.9 pA/pF (*n* = 9) in males.

## Discussion

### Sex Differences in Brain Development and Pathology

Sex differences are now recognized in brain development, adult brain structure and chemistry (Cosgrove et al., 2007); however, little is known about sex differences in specific brain cells. Recent studies have focused on how developmental disruptions might interfere with sex differentiation in the brain and lead to sex differences in later disease prevalence (Manoli and Tollkuhn, 2018). For instance, infant males are more prone to perinatal stroke, cerebral palsy, and to later development of autism spectrum disorders, attention deficit hyperactivity disorder, Tourette’s syndrome, early onset schizophrenia, amyotrophic lateral sclerosis, and Parkinson’s disease (Yanguas-Casas, 2017; Mallard et al., 2018). In many species, females have stronger immune systems and reduced susceptibility to infection and disease (Hanamsagar and Bilbo, 2016) but they are at risk of developing hyperactive immune systems and are more prone to developing multiple sclerosis, mood-related disorders, and Alzheimer’s disease (Yanguas-Casas, 2017). Early sex differences in the prevalence of CNS disorders are established before sexual maturation and adult circulating levels of sex hormones are attained. For this reason, we compared neonatal and prepubertal microglia.

The classical ‘organization-activation’ hypothesis posits that perinatal exposure to sex hormones organizes tissues (including sex-specific brain circuitry) such that a secondary exposure at maturation activates sexual dimorphisms (Arnold, 2009). In the perinatal male brain, there is a surge of testosterone, which is aromatized to estradiol (McCarthy, 2008). Sex-related behavioral differences can emerge at a very young age. For instance, in human infants, males have better object tracking abilities, while females react more intensely to painful stimuli (Manoli and Tollkuhn, 2018). Abnormal exposure to sex hormones early in development can have a profound effect on subsequent behavior. Female mice treated with estradiol at birth later exhibit male levels of territorial aggression (Wu et al., 2009). At the cellular level, the perinatal testosterone surge results in elevated numbers of neural progenitor cells in the hippocampus of male rats at birth; however, this difference is abolished if males are treated with an estrogen receptor antagonist (or aromatase inhibitor) or if females are treated with estradiol (Bowers et al., 2010). Most research has focused on organizational roles of sex hormones during development but differences also arise from sex chromosomes and gene expression (Hanamsagar and Bilbo, 2016; Nelson and Lenz, 2017).

### Sex Differences in Developing Microglia

There is increasing interest in potential sex differences in microglia, owing to their well-established roles in the CNS immune response and recent evidence for non-immunological roles in fine-tuning and maintaining neural circuitry (Nelson et al., 2017; Sominsky et al., 2018). Many studies investigate *in vitro* properties of neonatal microglia and there is increasing interest in possible sex differences in microglia during development and after CNS injury (Colonna and Butovsky, 2017; Dotson and Offner, 2017). The motivation for the present study was that early developmental differences between the sexes (testosterone surge, chromosomal differences) might influence their transcriptional and functional responses to well-known activating stimuli. To this end, we compared neonatal (P1) and prepubertal (P21) microglia of both sexes, with or without exposure to the pro-inflammatory cytokines, IFNγ + TNFα (I+T) or the anti-inflammatory cytokine, IL-4.

One functional sex difference we observed was in migration and its modulation by IL-4. IL-4 increases the migratory capacity of neonatal rat (Lively and Schlichter, 2013) and mouse microglia (Lam et al., 2017) in mixed-sex cultures. Here, IL-4 increased migration to a greater extent in male microglia, especially at P1. Greater migration of unstimulated neonatal male rat microglia was recently observed (Yanguas-Casas et al., 2018). These findings might help explain several *in vivo* observations. In male mice, microglia/macrophages began to surround the lesion earlier after traumatic brain injury (Villapol et al., 2017) and after a stab wound, which correlated with better preservation of neuron density (Acaz-Fonseca et al., 2015). Male mice also had more infiltrating immune cells after transient ischemia, and this difference was lost in IL-4 knockout mice (Xiong et al., 2015). Thus, IL-4 appears to play an important role in microglial migration and accumulation of inflammatory cells at sites of injury; responses that are greater in males. Many species, from insects to humans, display sexually divergent immune responses (Klein and Flanagan, 2016). Thus, we were surprised to find no sex-related differences in expression or responses of the >50 inflammation-related genes examined. Here, we will address several possible explanations.

(1) It is possible that the genes we examined are not regulated in a sex-dependent manner, under the activation conditions used, or at the ages examined. Consistent with our results, a recent study of embryonic, postnatal and adult male and female mouse hippocampal microglia found that basal gene expression was similar during development, and that sex differences only emerged at maturity (Hanamsagar et al., 2017). In adult mouse microglia, sex differences were seen in basal gene expression, and 37% of such genes were related to inflammation (Villa et al., 2018). Adult female mouse microglia had higher expression of inflammatory genes, especially those regulated by IFNγ (Thion et al., 2018); while male mouse microglia had higher NF-κB activity and expression of genes involved in cytokine secretion and migration (Villa et al., 2018). After CNS damage, some studies have reported a lack of sex differences in inflammatory responses; e.g., at 1 week after partial sciatic nerve ligation or traumatic brain injury in mice (Bruce-Keller et al., 2007; Lopes et al., 2017). However, others have noted sexual dimorphisms; e.g., at P3, female mouse microglia had higher basal expression of *Il1b*, *Il6*, *Il10*, *Tnf* (Crain et al., 2013) and the purinergic receptors, *P2rx5* and *P2ry4* (Crain et al., 2009). The potential for species differences needs to be addressed in future. We found that rat and mouse microglia differ in gene-expression profiles, both under baseline conditions and in response to pro- (I+T) and anti-inflammatory (IL-4, IL-10, TGFβ1) stimuli (Lam et al., 2017; Lively et al., 2018). Responses will also depend on the stimulus used. IL-4, IL-10 and TGFβ1 evoke different gene expression changes within a rodent species (Lam et al., 2017; Lively et al., 2018), and I+T evokes different responses from LPS (Lively and Schlichter, 2018). Not all results are consistent in the literature. In neonatal rat microglia, LPS evoked a higher increase in *Il1b* in males in one study (Loram et al., 2012) but not in another (Turano et al., 2017). However, in those two studies, the decrease in *Tlr4* was equal in both sexes (Loram et al., 2012; Turano et al., 2017), as it was after I+T in the present study. At puberty, a second surge of sex hormones activates many sexual dimorphisms throughout the body (Arnold, 2009), and it is possible that sex differences in microglial responses will emerge in microglia from older animals.

(2) Microglial gene expression might be regulated in a region-specific manner (we isolated microglia from whole brains, except the cerebellum) or depend on cell-cell interactions and the chemical milieu of the brain, which are lost after cell isolation. Most sex studies have focused on the intact brain, assessing cell numbers, morphology and basal gene expression (Nelson and Lenz, 2017). Before birth, microglial numbers and morphology were comparable between the sexes in the rat cortex, hippocampus and amygdala (Schwarz and Bilbo, 2012). However, at birth, females had a transient increase in amoeboid microglia in the amygdala, paraventricular nucleus and hippocampal CA3 region, which might be the result of increased volume in those regions in females. At P4, males had more amoeboid microglia in the amygdala, hippocampus and parietal cortex. Another study found that, at P3, male rats had more microglia overall, and more amoeboid cells in the preoptic area of the hypothalamus; treating females with estradiol at birth increased microglial number to male levels (Lenz et al., 2013). Not all results are consistent, as sex-related differences in these parameters were not seen at P3 in the rat hippocampus (Nelson et al., 2017). Instead, females had more microglia bearing phagocytic cups, and this was abrogated by estradiol treatment. Regional differences in gene expression have also been reported. At birth, male rat hippocampal and cortical tissue had higher expression of *Ccl4* and *Ccl20*, while females had higher *Il10*, *Il10ra* and *Il1r1* (Schwarz and Bilbo, 2012) but the cellular source of these immune mediators was not determined. Human microglia and whole cortical tissue show strikingly different gene expression profiles (Galatro et al., 2017), which might be due, at least in part, to astrocytes, which are also immunocompetent cells (Dong and Benveniste, 2001).

(3) Sex differences were not seen in baseline (control) gene expression or after I+T or IL-4 treatment. However, we examined only a single, 24 h time point. It is possible that sex differences in these responses occur at other times, whether later or transiently at earlier times.

### Age Differences in Microglia

As noted above, the rodent brain undergoes substantial developmental changes in the first 3 weeks after birth. Between P1 and P21, we observed many differences in baseline gene expression in rat microglia; and some are consistent with their known developmental roles. Neonatal (P1) microglia had higher levels of phagocytosis- (*Mrc1*, *Cd68*, *Pparg*, *Trem2*) and endocytosis- (*Tfrc*) related genes, as expected for their greater phagocytic activity (Li and Barres, 2018). Consistent with these results, neonatal mouse microglia have prominent phagocytic cups and enrichment of phagocytosis-related genes (Attaai et al., 2018). Prepubertal (P21) microglia had higher expression of some genes that are characteristic of mature microglia, including sensome-related molecules (*Csf1r*, *Cx3cr1*, *Itgam*, *Kcna3*, *P2ry12*), pro-inflammatory (*Casp1*, *Ifngr2*, *Il6*, *Ptgs2*, *Pycard*) and anti-inflammatory genes (*Ccl22*, *Cd163*, *Il10*, *Il10ra*), and immune modulators (*Nfkbia*, *Tlr2*). Similarly, in mouse microglia, expression of molecules related to pro-inflammatory responses becomes more prominent the third week of postnatal development (Attaai et al., 2018). Previous studies comparing microglia from embryonic, early postnatal and adult mice have also reported increased expression of many sensome-related genes at maturity in both sexes (Hanamsagar et al., 2017; Thion et al., 2018). The increases in sensome molecules we observed at P21 suggest that they are better primed to respond to disturbances. Pronounced apoptosis of microglia starts in the third postnatal week and is needed to achieve their adult numbers (Nikodemova et al., 2015); thus, the higher expression of immune-related molecules might be a response to microglial apoptosis. Importantly, over half the genes we examined (including all four categories) had comparable baseline levels at both ages; thus, P21 microglia are not simply more ‘activated.’ In future studies, it will be important to consider consequences of differences in specific genes. It is worth noting that there might also be species differences; e.g., *Nos2* and *Arg1* expression were very low at P1 in rat microglia (Lam et al., 2017; Lively et al., 2018); present study) but they peaked at P3 in mouse (Crain et al., 2013).

Age-dependent differences in gene expression can influence microglial responses to CNS insults and, conversely, brain insults can affect microglia development and their subsequent responses (Hanamsagar and Bilbo, 2017). Here, we will focus on a few molecules for comparison with the literature. Some responses were similar at P1 and P21. I+T increased expression and IL-4 decreased expression of *Aif1* and some pro-inflammatory mediators (*Hmox1*, *Pyk2*), but also several receptors (*Il10rb*, *Tgfbr1*, *Tnfrsf1a*, *Tnfrsf1b*) that suggest a priming toward subsequent resolution of pro-inflammatory responses. IL-4 increased the pattern recognition receptor, *Tlr4*, while I+T decreased it, consistent with IL-4 priming for subsequent innate immune responses. Many age-dependent differences were seen in response to the pro- and anti-inflammatory stimuli. I+T-mediated changes seen only at P1 include decreases in *Cd68*, *Pparg* and *Csf1*, and increases in several genes; i.e., some pro-inflammatory interleukins (*Il1a*, *Il1b*, *Il1r2*, *Il6*), anti-inflammatory molecules (*Arg1*, *Ccl22*, *Il13ra1*), and purinergic receptors (*P2rx4*, *P2rx7*, *P2ry2*). Only at P1 did IL-4 increase the protease activated receptor 1 (PAR-1/*F2r*), which is interesting because it is up-regulated in mouse microglia after intracerebral hemorrhage and contributes to edema, pro-inflammatory cytokine production and neuron death (Wan et al., 2016). A few changes were only seen at P21; e.g., I+T-mediated increases in *Cd68* and *Kcnn3* and decreases in *P2rx7* and *F2r*; and the IL-4-mediated increase in *Cd163* and decreases in *Csf1r*, *Cxcl10*, *Il1b* and *Nfkbia*. In addition, I+T evoked greater increases in *Cd274*/PD-L1 and *Cybb* at P1 but higher *Cxcl10* at P21. PD-L1 is interesting because LPS and IFNγ greatly increase it in mouse peritoneal macrophages (Loke and Allison, 2003), while CXCL10 propagates pro-inflammatory responses and promotes peripheral cell recruitment into the CNS (Scolletta et al., 2013). Overall, P21 microglia had higher basal and stimulated levels of many inflammatory genes, while P1 cells had higher expression of phagocytosis-related molecules.

### Kv and Kir Currents

Microglia express several K^+^ channels and, based on both *in vitro* and *in vivo* studies, Kv1.3 and is being considered as therapeutic targets to control CNS inflammation (recently reviewed in Peng et al., 2014; Feske et al., 2015). Most work that is relevant to the present study on Kv and Kir has used neonatal rodent cells *in vitro* to measure the currents and delineate roles for the channels. There appears to be no previous information on these channels in sex-segregated microglia. The only sex-relevant study we found was a report that estrogen inhibited the Kir2.1 current in the BV2 microglial cell line (Wu et al., 2016). For neonatal rats, we previously used combined-sex microglia, both unstimulated and cytokine-treated; and characterized Kv1.3 (e.g., Kotecha and Schlichter, 1999; Lam et al., 2017; Lively et al., 2018) and Kir2.1 currents (Lam and Schlichter, 2015; Lam et al., 2017; Lively et al., 2018). Most of those studies used selective blockers to isolate Kv1.3 and Kir2.1 currents from other potential K^+^ currents. Studies have begun to assess whether microglial K^+^ currents and their roles are activation-state dependent. Functionally, Kir2.1 regulates Ca^2+^ entry (Franchini et al., 2004; Lam and Schlichter, 2015), which is important for many cell functions, and blocking Kir2.1 reduced migration with or without microglial stimulation by IL-4, IL-10 or I+T (Franchini et al., 2004; Lam and Schlichter, 2015). Kv1.3 blockers decreased Ca^2+^ entry (Feske et al., 2015), NFκB activation, production of pro-inflammatory mediators, and microglia-mediated neuron killing (Khanna et al., 2001; Fordyce et al., 2005) but increased migration, regardless of the activation state (Lam et al., 2017). While Kv1.3 block did not affect phagocytosis of myelin in rat microglia (Siddiqui et al., 2016), it reduced phagocytosis of fluorescent beads in mouse microglia (Grimaldi et al., 2018). Neuroprotective effects of Kv1.3 blockers have been extended to *in vivo* injury models. For instance, Kv1.3 blockers reduced the infarct size after ischemia (Chen et al., 2018), radiation-induced brain injury (Peng et al., 2014), Alzheimer’s symptoms in mouse models (Maezawa et al., 2018), and microglia infiltration into glioblastoma tumors (Grimaldi et al., 2018).

There is still uncertainty about potential developmental regulation of Kv and Kir currents in microglia, and there appear to be species differences (Lam et al., 2017; Nguyen et al., 2017; Lively et al., 2018). Here, we were interested in the total Kv current that can contribute to cell function; rather than separating the specific Kv1.3 component, which is more relevant to drug targeting. No separation was needed for Kir because the total inward current in rat microglia is almost entirely Kir2.1 (Lam and Schlichter, 2015; Lam et al., 2017; Lively et al., 2018). In fact, most studies report Kv and Kir currents in neonatal rodent microglia. However, we did not find any patch-clamp studies on P21 or similar-aged prepubertal microglia, so comparisons will mainly concern neonatal rodent microglia. [It is worth noting that Kv and Kir currents have been reported in adult rodent microglia following acute CNS injury *in situ* (e.g., Lyons et al., 2000; Menteyne et al., 2009) but some studies have not observed them (e.g, Schilling and Eder, 2007; Arnoux et al., 2013; Chen et al., 2016). In neonatal rat microglia, K^+^ current amplitudes can change with cell activation states. Kv1.3 current was increased by I+T and IL-4 in rat and mouse microglia; whereas, Kir2.1 was decreased by IL-4 in rat and by I+T and IL-10 in mouse (Lam et al., 2017). TGFβ1 increased the Kv1.3 current in both species but did not affect Kir2.1 (Lively et al., 2018). Here, we addressed potential sex differences in Kv and Kir currents in P21 microglia, with and without stimulation by I+T or IL-4. Minor sex differences were seen. In response to IL-4, the Kir current appeared to increase slightly more in females but did not reach statistical significance for the sample size used. Although I+T increased Kv1.3 and Kir2.1 transcript expression in both sexes, the Kv current was increased much more in males. It remains to be determined whether Kv current sex differences under pro-inflammatory conditions occur *in vivo* and affect the ability of Kv1.3 blockers to ameliorate disease outcomes.

## Conclusion and Future Directions

It is increasingly recognized that microglia are malleable in their responses to insults *in vitro* and *in vivo*. If transcript expression and cytokine-evoked changes in the genes and functions we examined were ‘hard-wired’ during development or by the sex chromosomes, we would expect they would be maintained *in vitro*. However, epigenetic responses to the brain’s chemical milieu are expected to be more malleable and might revert after isolating the cells, which would be a specific limitation of *in vitro* studies. Even microglia development *in situ* can be altered by systemic (e.g., peripheral infection, gut microbiome) and environmental factors (e.g., pollution) (Hanamsagar and Bilbo, 2017). The present study assessed a targeted panel of genes to create a fingerprint of their initial state as well as responses to pro- and anti-inflammatory stimuli. Despite culturing the cells, we observed numerous age-dependent differences in basal and stimulated gene expression that are consistent with stable programming. Overall, both P1 and P21 cells were quite responsive to IL-4 and I+T. The minor sex differences observed — male microglia had greater migration after IL-4 and greater increases in Kv1.3 mRNA (*Kcna3*) and Kv current after I+T — raise the possibility that the testosterone surge had selective and enduring effects on the male brain. The surprising lack of sex differences in the genes we examined suggests they were either absent or that innate differences were not sustained during culture. The present results provide a framework for further investigating pro- and anti-inflammatory responses and it will be important to extend the study to *in vivo* damage models.

## Data Availability

The raw data supporting the conclusions of this manuscript will be made available by the authors, without undue reservation, to any qualified researcher.

## Author Contributions

SL and LS were conceived the project and wrote the manuscript. SL performed the NanoString sample collection, Western Blotting, staining, migration and Griess assays, and associated data analysis. RW and DL performed the patch-clamping and analysis.

## Conflict of Interest Statement

The authors declare that the research was conducted in the absence of any commercial or financial relationships that could be construed as a potential conflict of interest.

## Footnotes

1 http://www.transnetyx.com/

2 https://www.pmgenomics.ca/pmgenomics
