# Supplementary material for: Sex- and Development-Dependent Responses of Rat Microglia to Pro- and Anti-inflammatory Stimulation
Source: Front Cell Neurosci. 2018 Nov 20;12:433. doi: 10.3389/fncel.2018.00433 (PMC6262307; doi:10.3389/fncel.2018.00433)
Supplement: Supplementary file 1 [file Data_Sheet_1.PDF]

Sex- and development-dependent responses of rat microglia to pro- and anti-inflammatory stimulation

Starlee Lively, Raymond Wong, Doris Lam, and Lyanne C. Schlichter
Correspondence: Professor Lyanne C. Schlichter Lyanne.Schlichter@uhnresearch.ca or Dr. Starlee Lively Starlee.Lively@uhnresearch.ca

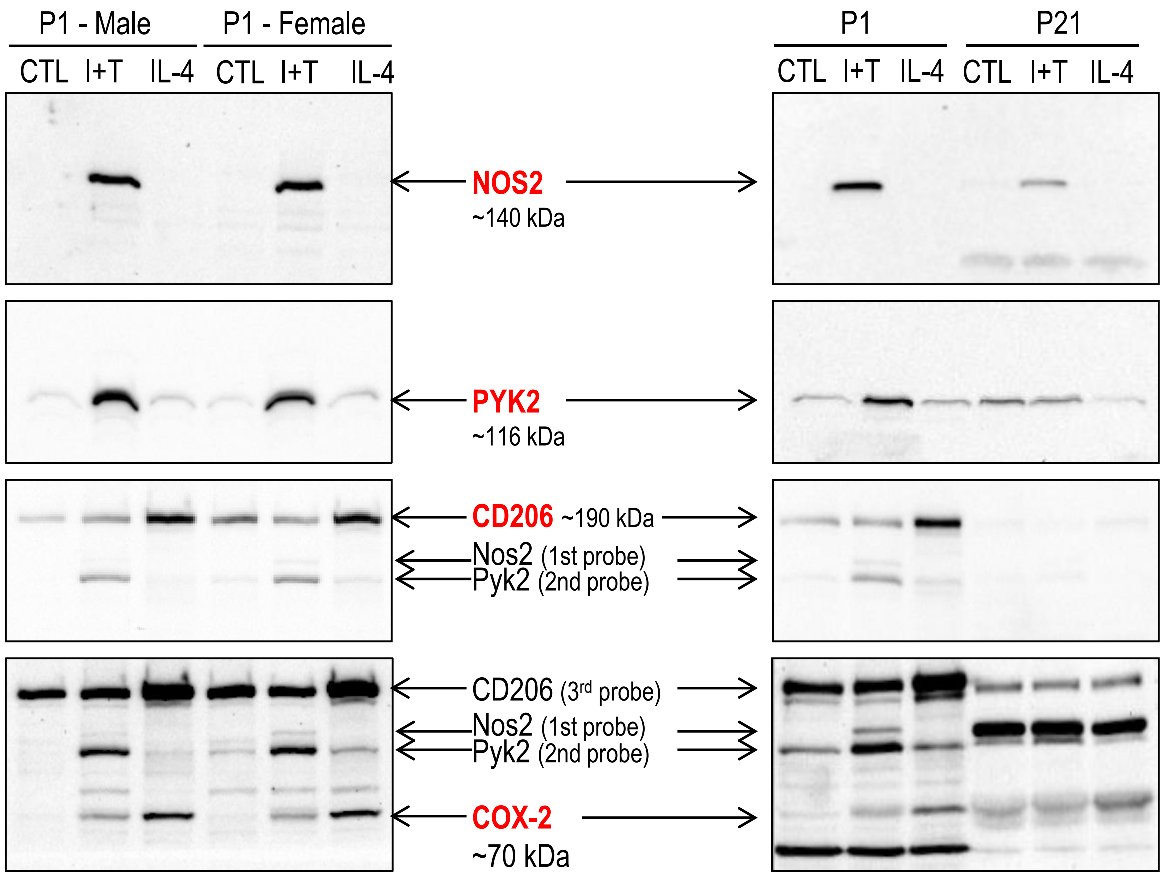

Supplementary Figure 1. Examples of Western Blots used for quantification of selected proteins at P1 and P21. Because samples were limited and molecular weights were similar for the proteins examined, blots were re-probed with additional antibodies. However, the protein bands of interest (red text) were easily isolated for quantification. The COX-2 antibody was always used last because it labeled several non-specific bands, and it labeled different bands at P1 and P21. At P1, the expected 70 kDa COX-2 band was readily identified and quantified. At P21 only, there was a prominent, non-specific band at ~140 kDa (i.e., near the NOS2 band) and because the 70 kDA band was unclear, COX-2 was not quantified at P21.

Sex- and development-dependent responses of rat microglia to pro- and anti-inflammatory stimulation

Starlee Lively, Raymond Wong, Doris Lam, and Lyanne C. Schlichter*
* Correspondence: Professor Lyanne C. Schlichter Lyanne.Schlichter@uhnresearch.ca or
Dr. Starlee Lively Starlee.Lively@uhnresearch.ca

Supplementary Table 1. Custom CodeSet target sequences for nCounter Assay

Gene	Accession #	Target sequence
Aif1	NM_017196.2	ATCGATATTATGTCCTTGAAGCGAATGCTGGAGAAACTTGGGGTTCCCAAGA CCCATCTAGAGCTGAAGAAATTAATTAGAGAGGTGTCCAGTGGCTCCG
Arg1	NM_017134.2	ACGGGAAGGTAATCATAAGCCAGAGACTGACTACCTTAAACCACCGAAATAA ATGTGAATACATCGCATAAAAGTCATCTGGGGCATCACAGCAAACCGA
Casp1	NM_012762.2	AGATTCTAAGGGAGGACATCCTTTCTCCTCAGAAACAAAAGAAAAACTGAAC AAAGAAGGTGGCGCATTTCCTGGACCGAGTGGTTCCCTCAAGTTTTGC
Ccl22	NM_057203.1	TACATCCGTCACCCTCTGCCACCACGTTTCGTGAAGGAGTTCTACTGGACCT CAAAGTCCTGCCGCAAGCCTGGCGTCGTTTTGATAACCATCAAGAACC
Cd163	NM_001107887.1	CCTCTGTAATTTGCTCAGGAAACCAATCGCATACACTGTTGCCATGTAGTTC ATCATCTTCGGTCCAAACAACAAGTTCTACCATTGCAAAGGACAGTGA
Cd200r1	NM_023953.1	CTGCTTTTGGAGAACTTCTCACGTAGCAGTACTCTTGATCTGGGGGGTCTTC GCGGCTGAGTCAAGTTGTCCTGATAAGAATCAAACAATGCAGAACAAT
Cd274	NM_001191954.1	CTGGGTACTCCTGGGATCCGTCCTTTTGTTCCTCATCGTGGGGTTCACCGTC TTCTTCTGCTTGAGAAAACAAGTGAGAATGCTAGATGTGGAAAAATGC
Cd68	NM_001031638.1	CTCTCATTCCCTTACGGACAGCTTACCTTTGGATTCAAACAGGACCGACATC AGAGCCACAGTACAGTCTACCTTAACTACATGGCAGTGGAATACAATG
Csf1	NM_023981.4	AGCCACATGATTGGGAATGGACACCTACAGATTTTGCAGCAGTTGATCGACA GCCAAATGGAGACTGCATGCCTGATCGAATATAAATTTGTAGACCAGG
Csf1r	NM_001029901.1	CCATGAACTTCCAGGTGGTGGAGAGTGCCTACTTAAACTTGACCTCTGAGCA GAGCCTCTTGCAGGAGGTGTCTGTGGGTGAGAACCTCGACCTCACAGT
Cx3cr1	NM_133534.1	ATGTGCAAGCTCACGACTGCTTTCTTCTTCATTGGCTTCTTTGGGGGCATAT TCTTCATCACCGTCATCAGCATCGACCGGTACCTCGCCATCGTCCTGG
Cxcl10	NM_139089.1	CTTCCCAATTCTCTAAGAGCTGGTCCGAATCTTCCCTCAGGCAGCTATGACG GCTCTCCTAGCTCTGTTCCGTAAGCTATGTGCAGGTACTAATCTCTTC
Cybb	NM_023965.1	CAGTACCAAAGTTTGCCGGAAACCCTCCTATGACTTGGAAATGGATCGTGGG TCCCATGTTCCTGTATCTGTGTGAGAGGCTGGTGCGGTTTTGGCGATC
F2r	NM_012950.2	CTGTAGAATAGCTTCCATGAAACTCCCCTAGTTGTCTGGTTAACTCTGTTCC TGTGTTGATTTATCTAAATCATTGACTCCCTGTCCTGTGCTCTGTGAC
Gapdh	NM_017008.2	TGGTGAAGGTCGGTGTGAACGGATTTGGCCGTATCGGACGCCTGGTTACCAG GGCTGCCTTCTCTTGTGACAAAGTGGACATTGTTGCCATCAACGACCC
Gusb	NM_017015.2	TCATTTGATCCTGGATGAGAAACGAAAAGAATATGTCATCGGAGAGCTCATC TGGAATTTTGCTGACTTCATGACGAACCAGTCACCACTGAGAGTAACA
Hmox1	NM_012580.2	CTAGTTCATCCCAGACACCGCTCCTGCGATGGGTCCTCACACTCAGTTTCCT GTTGGCGACCGTGGCAGTGGGAATTTATGCCATGTAAATGCAGTGTTG
Hprt1	NM_012583.2	AGCTTCCTCCTCAGACCGCTTTTCCCGCGAGCCGACCGGTTCTGTCATGTCG ACCCTCAGTCCCAGCGTCGTGATTAGTGATGATGAACCAGGTTATGAC
Ifnb1	NM_019127.1	CTCAGCTACAGGACGGACTTCAAGATCCCTATGGAGGTGATGCACCCGTCAC AGATGGAGAAGAGTTACACTGCCTTTGCCATTCAAGTGATGCTCCAGA
Ifng	NM_138880.2	AAGGACGGTAACACGAAAATACTTGAGAGCCAGATTATCTCTTTCTACCTCA GACTCTTTGAAGTCTTGAAAGACAACCAGGCCATCAGCAACAACATAA
Ifngr1	NM_053783.1	CCTGTTACACATTCGACTACACTGTGTTTGTGAAACATTACAGGAGTGGGGA GATCCTACATACAGAACATAGCGTCCTAAAAGAAGATTGTAGCGAAAC
Ifngr2	NM_001108313.1	TTTCTTAAGTTACACTTAGTAAAGCAGATGAGTCCGCAGGAGACTTCAGCAA GAAAGAAGTTCCTACCGTCTCATCCCTTAGTTCTTCAAAGCCAAAGGA

Il10	NM_012854.2	ACAACATACTGCTGACAGATTCCTTACTGCAGGACTTTAAGGGTTACTTGGG TTGCCAAGCCTTGTCAGAAATGATCAAGTTTTACCTGGTAGAAGTGAT
Il10ra	NM_057193.2	TGTTTACATGTCACGACGGAGCATTATTTCACCGTGACCAACCTCAGCATTT TCTTCTTATCCATCCTGATACTCTGTGGAGCCCTGGTCTGCCTGGTTC
Il10rb	NM_001107111.1	CCTCCCTGGATCGTGGCCATCATCCTTATAGCCTCCGTCTTGATAGTCTTCC TCTTCCTACTGGGCTGCTTCAGCATGGTGTGGTTCATTTACAAGAAGA
Il13	NM_053828.1	CTGTGCAGCCCTGGAATCCCTGACCAACATCTCCAGTTGCAATGCCATCCAC AGGACCCAGAGGATATTGAATGGCCTCTGTAACCAAAAGGCCTCGGAT
Il13ra1	NM_145789.2	TAACGAATTTGAGTGTCTCTGTCGAAAATCTCTGCACAATAGTGTGGACATG GAGTCCTCCTGAGGGAGCCAGTCCAAATTGCAGTCTCAGATATTTTAG
Il1a	NM_017019.1	TACTCATCGGGAGGAGACGACTCTAAATATCCTGTGACTCTCAAAGTCTCAA ATACTCAGCTCTTTGTGAGTGCTCAGGGAGAAGACAAGCCTGTGTTGC
Il1b	NM_031512.1	TGCACTGCAGGCTTCGAGATGAACAACAAAAATGCCTCGTGCTGTCTGACCC ATGTGAGCTGAAAGCTCTCCACCTCAATGGACAGAACATAAGCCAACA
Il1r1	NM_013123.3	CAGCGGACAAGGACTCCAGGATTCATCAGCAGAATGAACATCTTTGGTTTGT TCCCGCCAAGATGGAGGATTCAGGCTATTACTATTGTATAATGAGAAA
Il1r2	NM_053953.1	CCACTGTGAACAAATGTCTCTGGAACTCAAGGTCTTTAAGAATACCGAAGCC TCTTTCCCTCTCGTCTCCTACTTGCAAATCTCAGCTCTCTCCTCCACC
Il1rn	NM_022194.2	TCATTGCTGGGTACTTACAAGGACCAAATACCAAACTAGAAGAAAAGATAGA CATGGTGCCTATTGACTTTCGGAATGTGTTCTTGGGCATCCACGGGGG
Il4	NM_201270.1	TGCTGTCACCCTGTTCTGCTTTCTCATATGTACCGGGAACGGTATCCACGGA TGTAACGACAGCCCTCTGAGAGAGATCATCAACACTTTGAACCAGGTC
Il4r	NM_133380.2	GGGTGTCAGCATCTCCTGCATCTGCATCCTATTGTTTTGCCTGACCTGTTAC TTCAGCATTATCAAGATTAAGAAGATATGGTGGGACCAGATTCCCACT
Il6	NM_012589.1	GGAACAGCTATGAAGTTTCTCTCCGCAAGAGACTTCCAGCCAGTTGCCTTCT TGGGACTGATGTTGTTGACAGCCACTGCCTTCCCTACTTCACAAGTCC
Itgam	NM_012711.1	CATCCCTTCCTTCAACAGTAAAGAAATATTCAACGTCACCCTCCAGGGCAAT CTGCTATTTGACTGGTACATCGAGACTTCTCATGACCACCTCCTGCTT
Kcna3	NM_019270.3	GCCACCTTCTCCAGAAATATCATGAACCTGATAGACATTGTAGCCATCATCC CTTATTTTATTACTCTGGGCACTGAGCTGGCTGAGCGACAGGGTAATG
Kcna5	NM_012972.1	ATCAGAAGGGGTAGCTGTCCTCTAGAAAAGTGTCACCTCAAGGCCAAGAGCA ACGTGGACTTGCGGAGGTCCCTGTATGCCCTCTGTCTGGACACTAGCC
Kcnj2	NM_017296.1	GTTCTTTGGCTGTGTGTTTTGGTTGATAGCTCTGCTCCACGGGGATCTGGAT GCTTCTAAAGAGAGCAAAGCGTGTGTGTCTGAGGTCAACAGCTTCACG
Kcnn3	NM_019315.2	GTCATGCTGCTACATAGCAAGCTCTTCACGGATGCCTCATCCCGAAGCATCG GGGCCCTCAACAAGATCAACTTCAACACCCGATTCGTCATGAAGACGC
Kcnn4	NM_023021.2	TACGTCTCTACCTGGTGCCTCGCGCGGTACTTCTGCGTAGCGGGGTCCTGCT CAACGCGTCTTACCGCAGCATCGGGGCGCTCAACCAAGTCCGATTCCG
Lcn2	NM_130741.1	TACGATGTGCAAGTGGCCGACACTGACTACGACCAGTTTGCCATGGTATTTT TCCAGAAGACCTCTGAAAACAAACAGTACTTCAAAGTCACCCTGTACG
Mmp9	NM_031055.1	TGCGTCGGGCGCTGCTCCAACTGCTGTATAAATATTAAGGTATTCAGTTACT CCTACTGGAAGGTATTATGTAACCATTTCTCTCTTACATCGGAGGACA
Mrc1	NM_001106123.1	CTTTGGAATCAAGGGCACAGAGCTATATTTTAACTATGGCAACAGGCAAGAA AAGAATATCAAGCTTTACAAAGGTTCCGGTTTGTGGAGCAGATGGAAG
Msr1	NM_001191939.1	CACGTTCCATGACAGCATCCCTTCCTCACAACACTATAAATGGCTCCTCCGT TCAGGAGAAACTGAAGTCCTTCAAAGTTGCCCTCGTCGCTCTCTACCT
Ncf1	NM_053734.2	TCCATTCCCAGCATCCCATAATTGGGCTTGTCCGTGTTCCAACATCTGGGCG GAATTTCACAGCCAAAGGTCAAGAGGACTGCTGTTACGTTCAAGGTCG
Nfe2l2	NM_031789.1	ATACAACAAAAAAAGAAGTACCTGTGAGTCCTGGTCATCAAAAAGTCCCATT CACAAAAGACAAACATTCAAGCCGATTAGAGGCTCATCTCACAAGAGA
Nfkbia	XM_001075778.1	TATTGTGCTTTTGGTTGAACCGCCATAGACTGTAGCTGACCCCAGTGTGCCC TCTCACGTAAGAACCAGGTGTTCAGTGGTATGTGCTTAAGTCATCCCC
Nlrp3	NM_001191642.1	GTTCCAGAAGGAGGTGGACTGCGAGAGATTTTACAGCTTCAGCCACATGACT TTCCAGGAGTTCTTTGCGGCTATGTACTATCTGCTAGAAGAGGAGGAA
Nos2	NM_012611.2	ACGGGACACAGTGTCGCTGGTTTGAAACTTCTCAGCCACCTTGGTGAGGGGA

		CTGGACTTTTAGAGACGCTTCTGAGGTTCCTCAGGCTTGGGTCTTGTT
Nr3c1	NM_012576.2	AGCTTTCCTTGAAGCGTATAAAGAGCCATGCTCCTTTAGTATGTGGGGAAGA AGAGAGCTGTCATAGTTTTGAGTACAGTGAGAAGATGCGGTACTGTCT
P2rx4	NM_031594.1	GACCAGCATTTGTAATTCAGACGCCGACTGCACTCCTGGCTCCGTGGACACC CACAGCAGTGGAGTTGCGACTGGAAGATGTGTTCCTTTCAATGAGTCT
P2rx7	NM_019256.1	ACTTTAAGAGGTCACATTAACCAGACTAGAAGCCATCGCATCTAACCGCATA CCAGACACAGTCTGACGCCTCATTGCTATGCTATGGTTCTAAGTGACT
P2ry2	NM_017255.1	GAGCTCTTTAGCCATTTTGTGGCTTACAGCTCTGTCATGCTGGGTCTGCTTT TTGCTGTGCCCTTTTCCATCATCCTGGTCTGTTACGTGCTCATGGCCC
P2ry12	NM_022800.1	TGATAACCATTGACCGATACCTGAAGACCACCAGACCATTTAAAACTTCCAG CCCCAGCAATCTTTTGGGTGCGAAGATTCTTTCTGTTGCCATCTGGGC
Pdcd1	NM_001106927.1	CCACCTTCACCTGCAGTTTCTCCAACTGGTCGGAGGATCTTAAGCTGAACTG GTACCGTCTGAGTCCCAGCAACCAGACTGAAAAACAGGCCGCCTTCTG
Pparg	NM_013124.1	TTTATAGCTGTCATTATTCTCAGTGGAGACCGCCCAGGCTTGCTGAACGTGA AGCCCATCGAGGACATCCAAGACAACCTGCTGCAGGCCCTGGAACTCC
Pprc1	NM_001106363.1	CGTGCTCAGGGTTCTGAGGATGTGGTGCAGGCATTCATCAGTGAGATTGGGA TTGAAGCATCAGACCTGTCCAGTCTGTTGGAGCAGTTTGAGAAATCAG
Ptgs2	NM_017232.3	TTCGGAGGAGAAGTGGGTTTTAGGATCATCAACACTGCCTCAATTCAGTCTC TCATCTGCAATAATGTGAAAGGGTGTCCCTTTGCCTCTTTCAATGTGC
Ptk2b	NM_017318.2	GCAGTGATCATGAAGAATCTTGACCACCCTCACATCGTCAAGCTGATTGGCA TCATTGAAGAGGAACCCACATGGATCGTCATGGAACTGTATCCTTATG
Pycard	NM_172322.1	CTCAGGGCACAGCCAGAACAGAACATTTTGTGGACCAACACAGGCAAGCACT CATTGCCAGGGTCACAAAAGTTGATGGTTTGCTGGATGCTCTGTATGG
Retnla	NM_053333.1	AGGAACTTCTAGCCCATCAAGATAACTATCCCTCTGCTGTAAGGAAGACCCT CTCATGCACTAATGTCAAGTCTATGAGCAAATGGGCCTCCTGCCCTGC
Rpl32	NM_013226.2	CATCGTAGAAAGAGCAGCACAGCTGGCCATCAGAGTCACCAATCCCAACGCC AGGCTACGCAGCGAAGAGAATGAATAGATGGCTTGTGTGCCTGTTTTG
Sdha	NM_130428.1	CCTCCGATTAAGGCAAATGCTGGAGAAGAGTCGGTTATGAATCTTGACAAGT TGAGATTTGCTGATGGAAGTGTAAGAACATCAGAGCTGCGCCTCAGCA
Tfrc	XM_001072774.1	GCCAGATCAGCATTCTCTAACTTGTTTGGAGGGGAGCCATTGTCATACACCC GGTTTAGCCTTGCTCGGCAAGTAGATGGAGATAACAGTCATGTGGAGA
Tgfb1	NM_021578.2	CGCCTGCAGAGATTCAAGTCAACTGTGGAGCAACACGTAGAACTCTACCAGA AATATAGCAACAATTCCTGGCGTTACCTTGGTAACCGGCTGCTGACCC
Tgfbr1	NM_012775.2	GTCTGCATTGCACTTATGCTGATGGTCTATATCTGCCATAACCGCACTGTCA TTCACCACCGCGTACCAAATGAAGAGGATCCCTCACTAGATCGCCCTT
Tgfbr2	NM_031132.3	CCAGCAGTCCTGACCTGTTGCTGGTCATTATCCAAGTGACGGGCGTCAGCCT CCTGCCTCCGCTGGGGATTGCCATAGCTGTCATTGCCATCTTCTACTG
Tlr2	NM_198769.2	TTTACAAACCCTTAGGGTAGGAAATGTTGACACTTTCAGTGAGATAAGGAGA ATAGATTTTGCTGGGCTGACCTCTCTCAACGAACTTGAAATTCAGGTA
Tlr4	NM_019178.1	GTCAGTGTGCTTGTGGTAGCCACTGTAGCATTTCTGATATACCACTTCTATT TTCACCTGATACTTATTGCTGGCTGTAAAAAGTACAGCAGAGGAGAAA
Tnf	NM_012675.2	GGTGATCGGTCCCAACAAGGAGGAGAAGTTCCCAAATGGGCTCCCTCTCATC AGTTCCATGGCCCAGACCCTCACACTCAGATCATCTTCTCAAAACTCG
Tnfrsf1a	NM_013091.1	TATTCTTTATCTGCATCAGTCTACTGTGCCGATATCCCCAGTGGAGGCCCAG GGTCTACTCCATCATTTGTAGGGATTCAGCTCCTGTCAAAGAGGTGGA
Tnfrsf1b	NM_130426.4	AGGAGTTCAGATTCTTCCCATGGCAGCCACGGGACCCATGTCAACGTCACCT GCATCGTGAACGTCTGTAGCAGCTCTGACCACAGCTCTCAGTGTTCTT
Trem2	NM_001106884.1	TCCGGCTGGCTGAGGAAGGGTGCCATGGAACCTCTCCACGTGTTTGTCCTGT TGCTGGTCACAGAGCTGTCCCAAGCCCTCAACACCACAGTGCTGCAGG

Sex- and development-dependent responses of rat microglia to pro- and anti-inflammatory stimulation

Starlee Lively, Raymond Wong, Doris Lam, and Lyanne C. Schlichter
Correspondence: Professor Lyanne C. Schlichter Lyanne.Schlichter@uhnresearch.ca or Dr. Starlee Lively Starlee.Lively@uhnresearch.ca

Supplementary Table 2. Influence of sex on transcript levels of pro-inflammatory mediators in neonatal (P1) rat microglia

Microglia were isolated from 1 day-old male and female rat pups and stimulated with IFN-γ + TNF-α (I+T) or IL-4 for 24 h. For clarity, protein names are included for some genes. To show differences in basal mRNA levels, unstimulated (control) counts are expressed as mean counts/100 ng RNA sample ± SD (*n*=4–7 individual cultures). Effects of activation state on a given gene are expressed as fold-changes relative to sex-matched control levels; arrows represent significant increases (↑) or decreases (↓) in expression. One symbol indicates *p*<0.05; two, *p*<0.01; three, *p*<0.001. No sex differences within an activation paradigm were identified.

	Control		I+T		IL-4	
	mRNA counts ± SD		*Fold change with respect to Control± SD*			
	Male	Female	Male	Female	Male	Female
Casp1 (ICE)	133.94 ± 23.39	136.91 ± 34.92	2.06 ± 0.09 ↑↑	1.86 ± 0.38 ↑↑	0.88 ± 0.22	0.75 ± 0.16
Cd274 (PD-L1)	159.36 ± 37.91	158.80 ± 45.84	12.89 ± 2.04 ↑↑	14.95 ± 0.83 ↑↑	2.60 ± 0.98 ↑↑	2.00 ± 0.68 ↑↑
Cxcl10 (IP-10)	147.95 ± 79.76	214.55 ± 122.38	38.19 ± 5.27 ↑↑	20.57 ± 8.49 ↑↑	1.52 ± 1.47	0.62 ± 0.38
Ifnb1	1.76 ± 1.50	3.10 ± 3.05	3.37 ± 3.17	0.92 ± 0.62	2.79 ± 3.61	0.84 ± 0.68
Ifng	1.20 ± 0.26	2.37 ± 1.87	0.89 ± 0.15	0.45 ± 0.05	1.48 ± 1.23	0.61 ± 0.30
Ifngr1	2813.78 ± 390.78	2584.81 ± 303.72	0.54 ± 0.13 ↓↓	0.70 ± 0.23	0.36 ± 0.11 ↓↓	0.40 ± 0.12 ↓↓
Ifngr2	6.35 ± 3.78	9.64 ± 4.15	1.63 ± 0.55	1.57 ± 0.52	1.83 ± 0.86	1.19 ± 0.55
Il1a	81.58 ± 45.03	81.22 ± 42.42	5.89 ± 1.94 ↑↑	5.65 ± 1.65 ↑↑	5.26 ± 4.51 ↑	3.54 ± 3.64
Il1b	173.51 ± 91.79	185.16 ± 78.90	4.61 ± 1.55 ↑	4.17 ± 2.12	1.48 ± 1.33	0.77 ± 0.74
Il1r1	1.98 ± 1.33	3.57 ± 2.60	2.80 ± 1.71	0.94 ± 0.47	2.38 ± 2.66	1.58 ± 1.19
Il1r2	1.16 ± 0.17	1.45 ± 0.83	5.10 ± 4.41 ↑	3.27 ± 1.22 ↑	7.75 ± 5.93 ↑↑	11.06 ± 7.89 ↑↑
Il6	4.36 ± 3.81	1.52 ± 0.98	2.86 ± 1.58	9.46 ± 7.14 ↑	1.30 ± 1.52	7.06 ± 12.44
Nlrp3 (NALP3)	296.58 ± 81.78	304.61 ± 32.31	4.13 ± 1.39 ↑↑	4.15 ± 1.85 ↑↑	1.40 ± 0.69	0.92 ± 0.27
Nos2 (iNOS)	3.80 ± 4.06	5.14 ± 4.14	3081.04 ± 486.77 ↑↑	2362.11 ± 576.10 ↑↑	3.28 ± 3.72	1.76 ± 2.66
Ptgs2 (COX-2)	5.52 ± 5.84	4.04 ± 3.05	17.80 ± 2.91 ↑↑	17.45 ± 5.82 ↑↑	8.37 ± 12.10	11.28 ± 16.27
Ptk2b (PYK2)	376.19 ±36.59	440.27 ± 71.46	10.73 ± 1.18 ↑↑	9.27 ± 0.81 ↑↑	0.80 ± 0.31	0.60 ± 0.19 ↓↓
Pycard (ASC)	385.83 ± 90.48	402.60 ± 181.20	0.50 ± 0.04	0.49 ± 0.07	1.22 ± 0.42	0.99 ± 0.52
Tnf (TNF-α)	121.67 ± 85.27	126.44 ± 52.12	4.91 ± 1.14 ↑↑	4.93 ± 1.81 ↑↑	1.05 ± 0.52	0.62 ± 0.13
Tnfrsf1a (TNFR1)	291.94 ± 60.43	323.53 ± 22.84	2.88 ± 0.17 ↑↑	2.68 ± 0.34 ↑↑	0.78 ± 0.07 ↓↓	0.66 ± 0.06 ↓↓
Tnfrsf1b (TNFR2)	272.41 ± 55.11	304.18 ± 101.46	2.38 ± 0.48 ↑↑	2.37 ± 0.64 ↑↑	0.47 ± 0.16 ↓↓	0.40 ± 0.15 ↓↓

Sex- and development-dependent responses of rat microglia to pro- and anti-inflammatory stimulation

Starlee Lively, Raymond Wong, Doris Lam, and Lyanne C. Schlichter
Correspondence: Professor Lyanne C. Schlichter Lyanne.Schlichter@uhnresearch.ca or Dr. Starlee Lively Starlee.Lively@uhnresearch.ca

Supplementary Table 3. P1: Transcript expression of anti-inflammatory genes and receptors

Treatments, data presentation and analysis were as in Supplementary Table 2.

	Control		I+T		IL-4	
	mRNA counts ± SD		*Fold change with respect to Control± SD*			
	Male	Female	Male	Female	Male	Female
Arg1	1.39 ± 0.42	2.48 ± 1.75	3.39 ± 1.24 ↑	5.19 ± 2.35 ↑↑	10.44 ± 9.17 ↑↑	4.83 ± 2.33 ↑↑
Ccl22	1.39 ± 0.40	3.48 ± 2.77	4.33 ± 2.29	3.47 ± 1.52	133.27 ± 201.19 ↑↑	35.21 ± 50.74 ↑↑
Cd163	3.23 ± 2.96	3.46 ± 2.80	1.47 ± 0.77	0.85 ± 0.64	1.56 ± 0.81	1.66 ± 0.89
IL1rn (IL-RA)	267.87 ±186.87	638.08 ± 606.75	8.37 ± 1.62 ↑↑	4.58 ± 2.08 ↑↑	0.75 ± 0.53	0.27 ± 0.08
Il4	2.04 ± 2.13	4.04 ± 3.40	1.61 ± 1.40	0.41 ± 0.20	1.79 ± 3.15	0.71 ± 0.67
Il4r (IL-4RA)	175.25 ± 42.99	173.03 ± 15.26	3.84 ± 0.76 ↑↑	4.31 ± 1.65 ↑↑	1.13 ± 0.44	1.06 ± 0.47
Il10	1.20 ± 0.15	2.00 ± 1.65	0.91 ± 0.15	0.53 ± 0.05	1.00 ± 0.34	0.59 ± 0.22
Il10ra	167.93 ± 22.72	196.93 ± 42.39	4.59 ± 0.54 ↑↑	3.98 ± 0.70 ↑↑	0.96 ± 0.33	0.70 ± 0.17 ↓
Il10rb	340.12 ± 16.43	369.42 ± 30.06	1.50 ± 0.18 ↑↑	1.34 ± 0.24	0.55 ± 0.12 ↓↓	0.50 ± 0.09 ↓↓
Il13	1.48 ± 0.87	2.35 ± 2.43	0.99 ± 0.63	0.63 ± 0.31	1.67 ± 2.22	0.54 ± 0.19
Il13ra1	138.07 ± 18.85	147.35 ± 19.28	2.63 ± 0.26 ↑↑	2.55 ± 0.38 ↑↑	0.55 ± 0.15 ↓↓	0.55 ± 0.10 ↓↓
Mrc1 (CD206)	282.90 ± 40.27	275.30 ± 52.32	0.01 ± 0.01 ↓↓	0.01 ± 0.01 ↓↓	2.28 ± 0.65 ↑	2.46 ± 0.65 ↑↑
Pparg	23.98 ± 17.35	68.73 ± 62.18	0.17 ± 0.07	0.06 ± 0.04 ↓↓	1.67 ± 1.07	0.79 ± 0.64
Pprc1	43.12 ± 8.03	43.46 ± 9.42	0.95 ± 0.12	0.86 ± 0.32	1.58 ± 0.44	1.13 ± 0.27
Retnla (FIZZ1)	1.39 ± 0.47	5.05 ± 4.58	1.93 ± 1.22	0.26 ± 0.11	2.46 ± 3.61	0.47 ± 0.27
Tgfb1	3007.48 ± 1278.53	3223.42 ± 1199.60	0.60 ± 0.05	0.53 ± 0.11 ↓	1.10 ± 0.37	0.90 ± 0.25
Tgfbr1	1446.21 ± 242.81	1413.98 ± 260.64	0.48 ± 0.05 ↓↓	0.51 ± 0.06 ↓↓	0.43 ± 0.09 ↓↓	0.42 ± 0.08 ↓↓
Tgfbr2	412.76 ± 51.04	409.96 ± 51.64	1.19 ± 0.09	1.32 ± 0.31	0.49 ± 0.11 ↓↓	0.52 ± 0.12 ↓↓

Sex- and development-dependent responses of rat microglia to pro- and anti-inflammatory stimulation

Starlee Lively, Raymond Wong, Doris Lam, and Lyanne C. Schlichter
Correspondence: Professor Lyanne C. Schlichter Lyanne.Schlichter@uhnresearch.ca or Dr. Starlee Lively Starlee.Lively@uhnresearch.ca

Supplementary Table 4. P1: Transcript expression of selected microglia markers and immune modulators

Treatments, data presentation and analysis were as in Supplementary Table 2.

	Control		I+T		IL-4	
	mRNA counts ± SD		*Fold change with respect to Control± SD*			
	Male	Female	Male	Female	Male	Female
Aif (Iba1)	5204.37 ± 534.70	5516.37 ± 1240.64	1.88 ± 0.35 ↑↑	1.77 ± 0.38 ↑	0.59 ± 0.23 ↓↓	0.61 ± 0.26 ↓
Cd200r1	640.20 ± 412.42	685.14 ± 358.04	0.08 ± 0.01 ↓↓	0.08 ± 0.02 ↓↓	0.64 ± 0.27	0.53 ± 0.19
Cd68	12213.24 ± 1695.55	12684.43 ± 2405.68	0.60 ± 0.10 ↓↓	0.53 ± 0.13 ↓↓	0.49 ± 0.09 ↓↓	0.48 ± 0.13 ↓↓
Csf1	650.03 ± 257.69	678.53 ± 277.96	0.39 ± 0.06 ↓↓	0.47 ± 0.12 ↓	2.10 ± 0.78 ↑	1.67 ± 0.77
Csf1r	2190.31 ± 290.70	2439.41 ± 528.86	0.80 ± 0.12	0.79 ± 0.19	0.89 ± 0.16	0.88 ± 0.26
Cx3cr1	425.55 ± 153.49	329.29 ± 131.31	0.01 ± 0.01 ↓↓	0.02 ± 0.02 ↓↓	0.31 ± 0.22 ↓	0.43 ± 0.31
F2r (PAR-1)	15.17 ± 14.62	9.10 ± 5.20	0.35 ± 0.15	0.96 ± 0.41	8.53 ± 12.54 ↑↑	12.01 ± 9.62 ↑↑
Hmox1 (HO-1)	1703.47 ± 226.05	1738.47 ± 568.59	3.90 ± 0.70 ↑↑	3.58 ± 1.31 ↑↑	0.72 ± 0.49	0.67 ± 0.43
Itgam (CD11b)	948.16 ± 459.69	1010.94 ± 479.73	1.55 ± 0.25	1.43 ± 0.20	0.61 ± 0.39	0.53 ± 0.32
Lcn2 (NGAL)	7.24 ± 5.92	6.03 ± 3.53	1.16 ± 0.40	1.43 ± 0.27	1.06 ± 1.15	3.46 ± 4.93
Nfe2l2 (NRF2)	825.22 ± 96.50	1004.03 ± 198.64	2.64 ± 0.30 ↑↑	2.21 ± 0.33 ↑↑	0.95 ± 0.28	0.63 ± 0.13 ↓↓
Nfkbia (IκBα)	1149.76 ± 254.18	1153.23 ± 232.75	6.17 ± 0.92 ↑↑	5.99 ± 0.85 ↑↑	1.27 ± 0.71	0.75 ± 0.32
Nr3c1 (GR)	263.36 ± 11.73	255.16 ± 20.84	2.76 ± 0.56 ↑↑	2.78 ± 0.34 ↑↑	0.72 ± 0.14 ↓↓	0.65 ± 0.08 ↓↓
Pdcd1 (PD-1)	3.13 ± 2.05	4.24 ± 2.87	1.56 ± 1.04	1.44 ± 0.66	0.94 ± 1.03	0.66 ± 0.35
Tfrc (CD71)	278.90 ± 69.04	284.72 ± 99.61	0.79 ± 0.22	0.64 ± 0.09	1.26 ± 0.25	0.97 ± 0.26
Tlr2	1030.66 ± 255.52	964.98 ± 142.28	1.91 ± 0.47	1.90 ± 0.76	0.29 ± 0.13 ↓↓	0.27 ± 0.11 ↓↓
Tlr4	166.97 ± 49.87	169.10 ± 41.73	0.60 ± 0.15	0.56 ± 0.13 ↓	2.05 ± 0.66 ↑↑	1.88 ± 0.37 ↑↑
Trem2	2967.91 ± 614.30	2994.71 ± 569.13	0.04 ± 0.03 ↓↓	0.04 ± 0.01 ↓↓	0.27 ± 0.12 ↓↓	0.27 ± 0.08 ↓↓

Sex- and development-dependent responses of rat microglia to pro- and anti-inflammatory stimulation

Starlee Lively, Raymond Wong, Doris Lam, and Lyanne C. Schlichter
Correspondence: Professor Lyanne C. Schlichter Lyanne.Schlichter@uhnresearch.ca or Dr. Starlee Lively Starlee.Lively@uhnresearch.ca

Supplementary Table 5. P1: Transcript expression of genes related to microglial physiology
Treatments, data presentation and analysis were as in Supplementary Table 2.

	Control			I+T			IL-4	
	mRNA counts ± SD			*Fold change with respect to Control± SD*				
	Male	Female		Male	Female		Male	Female
Cybb (NOX2)	411.69 ± 145.20	473.38 ± 240.81		2.36 ± 0.31 ↑	2.22 ± 0.46 ↑		0.37 ± 0.19 ↓↓	0.35 ± 0.16 ↓↓
Kcna3 (Kv1.3)	17.20 ± 6.79	14.06 ± 5.90		2.59 ± 0.83 ↑↑	3.09 ± 1.29 ↑↑		0.94 ± 0.29	1.23 ± 0.29
Kcna5 (Kv1.5)	1.48 ± 0.87	3.79 ± 3.62		1.07 ± 0.61	0.28 ± 0.03		1.53 ± 1.87	0.53 ± 0.38
Kcnj2 (Kir2.1)	414.86 ± 238.40	443.00 ± 173.46		5.02 ± 0.63 ↑↑	5.28 ± 1.26 ↑↑		0.41 ± 0.09 ↓↓	0.30 ± 0.05 ↓↓
Kcnn3 (KCa2.3)	1.20 ± 0.26	3.98 ± 2.91		2.30 ± 1.79	1.14 ± 0.71		1.00 ± 0.34	0.53 ± 0.25
Kcnn4 (KCa3.1)	9.04 ± 5.96	14.16 ± 3.52		2.27 ± 0.87	1.97 ± 1.14		2.79 ± 2.28	1.79 ± 1.47
Mmp9	293.77 ± 256.16	237.88 ± 131.87		0.29 ± 0.10	0.75 ± 0.48		2.23 ± 2.23	2.43 ± 2.23
Msr1 (SR-A)	2452.06 ± 1587.45	2718.76 ± 1239.87		0.31 ± 0.12 ↓	0.27 ± 0.08 ↓↓		0.42 ± 0.2	0.38 ± 0.15 ↓
Ncf1	1844.46 ± 518.70	1710.31 ± 294.93		5.60 ± 2.20 ↑↑	4.97 ± 1.71 ↑↑		0.51 ± 0.19 ↓↓	0.43 ± 0.10 ↓↓
P2rx4	939.01 ± 292.71	885.24 ± 108.07		2.77 ± 0.54 ↑↑	2.91 ± 0.88 ↑↑		0.66 ± 0.15	0.59 ± 0.12 ↓↓
P2rx7	44.04 ± 16.93	54.73 ± 12.89		4.13 ± 2.57 ↑↑	3.48 ± 1.49 ↑↑		2.23 ± 0.58 ↑	1.43 ± 0.47
P2ry2	39.07 ± 27.04	45.70 ± 17.15		2.65 ± 0.75 ↑↑	2.58 ± 0.63 ↑↑		2.12 ± 1.26 ↑	1.82 ± 1.00
P2ry12	129.33 ± 48.62	131.18 ± 53.30		0.33 ± 0.12 ↓	0.36 ± 0.16 ↓		1.25 ± 1.15	1.44 ± 0.97

Sex- and development-dependent responses of rat microglia to pro- and anti-inflammatory stimulation

Starlee Lively, Raymond Wong, Doris Lam, and Lyanne C. Schlichter
Correspondence: Professor Lyanne C. Schlichter Lyanne.Schlichter@uhnresearch.ca or Dr. Starlee Lively Starlee.Lively@uhnresearch.ca

Supplementary Table 6. Influence of sex on transcript levels of pro-inflammatory mediators in microglia at time of weaning (P21)
Microglia were isolated from 21-day-old male and female rat pups and stimulated with IFN-γ + TNF-α (I+T) or IL-4 for 24 h. For clarity, protein names are included for some genes. To show differences in basal mRNA levels, unstimulated (control) counts are expressed as mean counts/100 ng RNA sample ± SD (*n*=4–7 individual cultures). Effects of activation state on a given gene are expressed as fold-changes relative to sex-matched control levels. Arrows represent significant increases (↑) or decreases (↓). One symbol indicates *p*<0.05; two, *p*<0.01; three, *p*<0.001. Sex differences within an activation paradigm were not detected.

	Control		I+T		IL-4	
	mRNA counts ± SD		*Fold change with respect to Control± SD*			
	Male	Female	Male	Female	Male	Female
Casp1 (ICE)	260.58 ± 100.78	276.70 ± 80.59	2.23 ± 0.45 ↑↑	2.29 ± 0.34 ↑↑	0.73 ± 0.22	0.61 ± 0.09
Cd274 (PD-L1)	274.88 ± 120.07	270.92 ± 149.35	4.78 ± 1.22 ↑↑	4.97 ± 0.95 ↑↑	1.18 ± 0.68	1.59 ± 0.75
Cxcl10 (IP-10)	767.39 ± 671.94	568.87 ± 402.54	16.99 ± 1.48 ↑↑	22.80 ± 4.72 ↑↑	0.25 ± 0.32	0.19 ± 0.19
Ifnb1	1.79 ± 0.10	4.18 ± 1.57	6.95 ± 3.81 ↑↑	1.63 ± 1.56	0.95 ± 0.37	0.49 ± 0.29
Ifng	1.79 ± 0.10	3.26 ± 1.68	1.16 ± 0.29	0.99 ± 0.66	0.86 ± 0.18	0.63 ± 0.37
Ifngr1	1668.98 ± 706.10	1822.83 ± 652.06	0.46 ± 0.02 ↓	0.41 ± 0.03 ↓↓	0.80 ± 0.41	0.60 ± 0.25
Ifngr2	28.68 ± 9.18	29.69 ± 17.84	1.43 ± 0.52	1.05 ± 0.66	0.84 ± 0.45	0.84 ± 0.44
Il1a	694.22 ± 668.32	780.74 ± 863.31	0.63 ± 0.43	0.42 ± 0.13	0.37 ± 0.21	0.49 ± 0.46
Il1b	1853.48 ± 1597.34	1516.36 ± 1396.97	1.03 ± 0.25	0.95 ± 0.30	0.21 ± 0.18	0.29 ± 0.31
Il1r1	13.27 ± 14.55	5.26 ± 2.15	1.26 ± 0.94	1.15 ± 1.00	0.79 ± 0.82	0.89 ± 0.78
Il1r2	12.02 ± 11.13	11.46 ± 11.56	0.90 ± 0.33	0.68 ± 0.56	0.55 ± 0.25	0.53 ± 0.43
Il6	59.63 ± 60.03	36.44 ± 27.62	1.90 ± 1.16	1.84 ± 0.30	0.25 ± 0.16	0.48 ± 0.53
Nlrp3 (NALP3)	584.05 ± 186.45	577.15 ± 148.54	1.65 ± 0.26 ↑	1.93 ± 0.42 ↑↑	0.81 ± 0.23	0.78 ± 0.08
Nos2 (iNOS)	91.09 ± 97.83	54.03 ± 61.01	103.04 ± 62.47 ↑↑	164.5 ± 29.20 ↑↑	0.15 ± 0.08	0.46 ± 0.60
Ptgs2 (COX-2)	39.17 ± 33.05	18.42 ± 18.88	5.68 ± 0.91 ↑	13.00 ± 3.37 ↑↑	0.28 ± 0.08	1.10 ± 0.98
Ptk2b (PYK2)	662.25 ± 294.29	708.42 ± 316.05	5.07 ± 0.58 ↑↑	5.32 ± 0.72 ↑↑	0.53 ± 0.27	0.48 ± 0.27
Pycard (ASC)	776.71 ± 293.66	999.91 ± 252.46	0.60 ± 0.12	0.44 ± 0.06 ↓↓	1.04 ± 0.20	0.76 ± 0.16
Tnf (TNF-α)	252.09 ± 209.53	181.49 ± 135.49	2.92 ± 0.47 ↑	3.83 ± 1.20 ↑↑	0.34 ± 0.16	0.59 ± 0.33
Tnfrsf1a (TNFR1)	334.12 ± 83.03	348.26 ± 58.06	3.01 ± 0.34 ↑↑	2.88 ± 0.24 ↑↑	0.78 ± 0.11	0.78 ± 0.17
Tnfrsf1b (TNFR2)	316.13 ± 151.47	312.34 ± 128.06	1.75 ± 0.55	1.77 ± 0.41	0.52 ± 0.28	0.53 ± 0.28

Sex- and development-dependent responses of rat microglia to pro- and anti-inflammatory stimulation

Starlee Lively, Raymond Wong, Doris Lam, and Lyanne C. Schlichter
Correspondence: Professor Lyanne C. Schlichter Lyanne.Schlichter@uhnresearch.ca or Dr. Starlee Lively Starlee.Lively@uhnresearch.ca

Supplementary Table 7. P21: Transcript expression of anti-inflammatory genes and receptors
Treatments, data presentation and analysis were as in Supplementary Table 6.

	Control		I+T		IL-4	
	mRNA counts ± SD		*Fold change with respect to Control± SD*			
	Male	Female	Male	Female	Male	Female
Arg1	1.79 ± 0.10	8.21 ± 6.62	6.51 ± 1.05 ↑	1.18 ± 1.35	3.61 ± 1.74	1.48 ± 2.22
Ccl22	46.90 ± 38.42	30.51 ± 12.61	0.79 ± 0.68	1.66 ± 0.85	0.32 ± 0.12	1.31 ± 1.40
Cd163	40.84 ± 61.65	26.99 ± 33.07	0.12 ± 0.08	0.37 ± 0.15	1.34 ± 0.95	2.43 ± 1.55
IL1rn (IL-RA)	531.84 ± 251.10	566.29 ± 192.81	5.86 ± 1.87 ↑↑	6.14 ± 2.49 ↑↑	0.63 ± 0.29	0.70 ± 0.29
Il4	2.41 ± 1.33	3.26 ± 1.68	1.53 ± 0.61	1.56 ± 0.77	0.82 ± 0.32	0.87 ± 0.59
Il4r (IL-4RA)	281.60 ± 138.03	216.28 ± 69.25	1.90 ± 0.44 ↑↑	2.28 ± 0.40 ↑↑	0.86 ± 0.23	0.96 ± 0.10
Il10	5.76 ± 6.54	6.46 ± 6.17	0.53 ± 0.42	0.59 ± 0.54	0.27 ± 0.06	0.56 ± 0.52
Il10ra	261.00 ± 84.76	293.83 ± 76.10	2.87 ± 0.65 ↑↑	2.80 ± 0.61 ↑↑	0.96 ± 0.19	0.87 ± 0.15
Il10rb	328.22 ± 107.51	361.31 ± 71.64	1.51 ± 0.21	1.44 ± 0.11	0.73 ± 0.17	0.67 ± 0.10
Il13	2.07 ± 0.58	3.41 ± 1.47	1.49 ± 1.18	1.12 ± 0.90	0.74 ± 0.16	0.60 ± 0.35
Il13ra1	176.16 ± 85.03	208.67 ± 105.89	1.39 ± 0.24	1.22 ± 0.30	0.50 ± 0.27	0.34 ± 0.11 ↓↓
Mrc1 (CD206)	99.25 ± 68.65	104.40 ± 83.46	0.02 ± 0.01 ↓↓	0.02 ± 0.01 ↓↓	1.47 ± 0.80	1.65 ± 0.56
Pparg	3.37 ± 1.61	3.41 ± 1.47	0.61 ± 0.16	1.50 ± 0.91	0.59 ± 0.26	1.43 ± 0.78
Pprc1	70.44 ± 27.48	62.75 ± 17.69	0.94 ± 0.12	1.09 ± 0.08	0.98 ± 0.17	1.12 ± 0.33
Retnla (FIZZ1)	2.04 ± 0.60	3.92 ± 1.31	1.87 ± 1.79	0.89 ± 0.41	1.13 ± 0.37	0.68 ± 0.26
Tgfb1	3134.67 ± 810.30	3349.10 ± 856.59	0.59 ± 0.05 ↓↓	0.54 ± 0.07 ↓↓	1.02 ± 0.11	1.12 ± 0.21
Tgfbr1	1386.49 ± 475.92	1723.32 ± 703.82	0.73 ± 0.22	0.55 ± 0.15	0.65 ± 0.36	0.51 ± 0.25
Tgfbr2	444.76 ± 184.83	446.37 ± 129.05	1.31 ± 0.21	1.23 ± 0.18	0.60 ± 0.12	0.65 ± 0.11

Sex- and development-dependent responses of rat microglia to pro- and anti-inflammatory stimulation

Starlee Lively, Raymond Wong, Doris Lam, and Lyanne C. Schlichter
Correspondence: Professor Lyanne C. Schlichter Lyanne.Schlichter@uhnresearch.ca or Dr. Starlee Lively Starlee.Lively@uhnresearch.ca

Supplementary Table 8. P21: Transcript expression of selected microglia markers and immune modulators

Treatments, data presentation and analysis were as in Supplementary Table 6.

	Control		I+T		IL-4	
	mRNA counts ± SD		*Fold change with respect to Control± SD*			
	Male	Female	Male	Female	Male	Female
Aif (Iba1)	5489.12 ± 1946.66	6189.06 ± 2004.63	1.74 ± 0.29	1.66 ± 0.20	0.78 ± 0.49	0.59 ± 0.31
Cd200r1	457.71 ± 144.96	516.57 ± 220.77	0.24 ± 0.13 ↓↓	0.17 ± 0.03 ↓↓	0.98 ± 0.23	1.05 ± 0.28
Cd68	3365.60 ± 726.52	4102.14 ± 789.86	1.37 ± 0.21	1.24 ± 0.19	0.88 ± 0.28	0.82 ± 0.13
Csf1	481.21 ± 567.72	438.90 ± 485.42	0.49 ± 0.33	0.34 ± 0.10	1.67 ± 0.93	2.65 ± 1.44
Csf1r	3334.08 ± 748.76	3538.54 ± 935.12	0.53 ± 0.04 ↓↓	0.47 ± 0.06 ↓↓	0.82 ± 0.16	0.70 ± 0.14
Cx3cr1	1693.39 ± 811.42	2173.62 ± 1064.17	0.19 ± 0.09 ↓	0.16 ± 0.10 ↓↓	0.74 ± 0.63	0.45 ± 0.46
F2r (PAR-1)	35.03 ± 40.67	18.75 ± 28.62	0.18 ± 0.17	0.21 ± 0.07	0.94 ± 0.56	2.43 ± 1.67
Hmox1 (HO-1)	2501.47 ± 1409.96	3110.59 ± 988.14	2.26 ± 0.69 ↑	1.95 ± 0.46	0.58 ± 0.23	0.58 ± 0.28
Itgam (CD11b)	3439.18 ± 1016.25	3438.99 ± 689.03	1.46 ± 0.32	1.32 ± 0.22	0.67 ± 0.41	0.63 ± 0.22
Lcn2 (NGAL)	192.18 ± 139.37	114.52 ± 125.20	1.55 ± 2.54	0.60 ± 0.42	0.31 ± 0.22	0.47 ± 0.34
Nfe2l2 (NRF2)	1150.79 ± 416.04	1201.70 ± 513.10	1.95 ± 0.42	1.84 ± 0.18 ↑	0.60 ± 0.34	0.54 ± 0.18
Nfkbia (IκBα)	3279.54 ± 1376.99	3498.0 ± 1078.69	2.13 ± 0.16 ↑	2.04 ± 0.19 ↑	0.59 ± 0.29	0.57 ± 0.23
Nr3c1 (GR)	306.02 ± 56.71	318.70 ± 61.25	2.00 ± 0.36 ↑↑	2.47 ± 0.53 ↑↑	0.93 ± 0.12	0.90 ± 0.14
Pdcd1 (PD-1)	2.29 ± 0.76	4.53 ± 1.87	3.11 ± 3.17	0.91 ± 0.72	1.46 ± 1.55	0.97 ± 1.29
Tfrc (CD71)	168.68 ± 75.83	136.33 ± 48.56	0.44 ± 0.47 ↓	0.34 ± 0.09 ↓	1.09 ± 0.34	1.33 ± 0.38
Tlr2	2743.05 ± 1404.66	2630.18 ± 1227.00	1.34 ± 0.10	1.42 ± 0.12	0.34 ± 0.35	0.29 ± 0.30 ↓
Tlr4	245.04 ± 123.10	268.77 ± 160.08	0.50 ± 0.12	0.50 ± 0.11	1.40 ± 0.55	1.51 ± 0.61
Trem2	950.48 ± 199.99	1015.54 ± 244.49	0.18 ± 0.17 ↓↓	0.10 ± 0.05 ↓↓	0.89 ± 0.31	0.80 ± 0.24

Sex- and development-dependent responses of rat microglia to pro- and anti-inflammatory stimulation

Starlee Lively, Raymond Wong, Doris Lam, and Lyanne C. Schlichter
Correspondence: Professor Lyanne C. Schlichter Lyanne.Schlichter@uhnresearch.ca or Dr. Starlee Lively Starlee.Lively@uhnresearch.ca

Supplementary Table 9. P21: Transcript expression of genes related to microglial physiology
Treatments, data presentation and analysis were as in Supplementary Table 6.

	Control		I+T		IL-4	
	mRNA counts ± SD		*Fold change with respect to Control± SD*			
	Male	Female	Male	Female	Male	Female
Cybb (NOX2)	305.07 ± 163.21	305.02 ± 116.77	1.89 ± 0.18	1.80 ± 0.27	0.52 ± 0.48	0.45 ± 0.35
Kcna3 (Kv1.3)	56.48 ± 27.96	56.29 ± 24.24	3.41 ± 1.44 ↑↑	2.62 ± 0.98 ↑	0.65 ± 0.18	0.59 ± 0.24
Kcna5 (Kv1.5)	3.38 ± 2.96	4.26 ± 2.24	0.91 ± 0.72	0.66 ± 0.19	0.66 ± 0.49	0.59 ± 0.42
Kcnj2 (Kir2.1)	585.97 ± 372.06	589.24 ± 268.80	4.63 ± 1.41 ↑↑	4.86 ± 1.11 ↑↑	0.53 ± 0.38	0.47 ± 0.16
Kcnn3 (KCa2.3)	3.17 ± 2.51	3.26 ± 1.68	1.99 ± 1.12	3.69 ± 3.31	0.53 ± 0.21	0.63 ± 0.37
Kcnn4 (KCa3.1)	15.99 ± 12.56	16.30 ± 12.76	1.71 ± 0.80	1.50 ± 0.54	0.75 ± 0.12	1.17 ± 0.64
Mmp9	143.02 ± 47.74	133.14 ± 93.77	1.10 ± 0.63	0.83 ± 0.42	0.66 ± 0.28	1.16 ± 0.80
Msr1 (SR-A)	4193.33 ± 1837.95	3783.79 ± 1373.80	0.38 ± 0.10 ↓	0.41 ± 0.13 ↓	0.57 ± 0.22	0.67 ± 0.23
Ncf1	3785.28 ± 1476.39	4949.72 ± 2269.11	2.52 ± 0.74	1.91 ± 0.63	0.60 ± 0.40	0.44 ± 0.31
P2rx4	764.61 ± 164.22	1191.96 ± 570.22	1.98 ± 1.05	1.22 ± 0.52	1.21 ± 0.49	0.78 ± 0.29
P2rx7	383.92 ± 28.89	489.79 ± 110.07	0.57 ± 0.06 ↓↓	0.56 ± 0.17 ↓↓	1.06 ± 0.15	0.94 ± 0.15
P2ry2	49.90 ± 19.70	45.67 ± 9.92	1.24 ± 0.34	1.43 ± 0.25	0.73 ± 0.52	1.11 ± 0.49
P2ry12	238.47 ± 125.38	304.16 ± 138.63	0.24 ± 0.09 ↓↓	0.16 ± 0.06 ↓↓	1.20 ± 0.36	0.73 ± 0.30

Sex- and development-dependent responses of rat microglia to pro- and anti-inflammatory stimulation

Starlee Lively, Raymond Wong, Doris Lam, and Lyanne C. Schlichter
Correspondence: Professor Lyanne C. Schlichter Lyanne.Schlichter@uhnresearch.ca or Dr. Starlee Lively Starlee.Lively@uhnresearch.ca

Supplementary Table 10. Age comparisons – Female. Transcript levels of pro-inflammatory mediators
Microglia were isolated from 1- and 21-day-old female rat pups and stimulated with IFN-γ + TNF-α (I+T), IL-4 or IL-10 for 24 h. For clarity, protein names are included for some genes. To show differences in basal mRNA levels, unstimulated (control) counts are expressed as mean counts/100 ng RNA sample ± SD (*n*=4–7 individual cultures). Effects of activation state on a given gene are expressed as fold-changes relative to age-matched control levels; arrows represent significant increases (red arrows) or decreases (blue arrows) in expression. Age differences within an activation paradigm are indicated by asterisks. One symbol (arrow or asterisk) indicates *p*<0.05; two, *p*<0.01; three, *p*<0.001

	Control		I+T		IL-4	
	mRNA counts ± SD		*Fold change with respect to Control± SD*			
	P1	P21	P1	P21	P1	P21
Casp1 (ICE)	136.91 ± 34.92	276.70 ± 80.59 **	1.86 ± 0.38 ↑↑	2.29 ± 0.34 ↑↑ **	0.75 ± 0.16	0.61 ± 0.09 ↓ **
Cd274 (PD-L1)	158.80 ± 45.84	270.92 ± 149.35	14.95 ± 0.83 ↑↑	4.97 ± 0.95 ↑↑	2.00 ± 0.68	1.59 ± 0.75
Cxcl10 (IP-10)	214.55 ± 122.38	568.87 ± 402.54	20.57 ± 8.49 ↑↑	22.80 ± 4.72 ↑↑	0.62 ± 0.38	0.19 ± 0.19 ↓
Ifnb1	3.10 ± 3.05	4.18 ± 1.57	0.92 ± 0.62	1.63 ± 1.56	0.84 ± 0.68	0.49 ± 0.29
Ifng	2.37 ± 1.87	3.26 ± 1.68	0.45 ± 0.05	0.99 ± 0.66	0.61 ± 0.30	0.63 ± 0.37
Ifngr1	2584.81 ± 303.72	1822.83 ± 652.06	0.70 ± 0.23	0.41 ± 0.03 ↓↓ **	0.40 ± 0.12 ↓↓	0.60 ± 0.25 ↓
Ifngr2	9.64 ± 4.15	29.69 ± 17.84	1.57 ± 0.52	1.05 ± 0.66	1.19 ± 0.55	0.84 ± 0.44
Il1a	81.22 ± 42.42	780.74 ± 863.31 **	5.65 ± 1.65 ↑↑	0.42 ± 0.13	3.54 ± 3.64	0.49 ± 0.46
Il1b	185.16 ± 78.90	1516.36 ± 1396.97 *	4.17 ± 2.12	0.95 ± 0.30	0.77 ± 0.74	0.29 ± 0.31
Il1r1	3.57 ± 2.60	5.26 ± 2.15	0.94 ± 0.47	1.15 ± 1.00	1.58 ± 1.19	0.89 ± 0.78
Il1r2	1.45 ± 0.83	11.46 ± 11.56 **	3.27 ± 1.22	0.68 ± 0.56	11.06 ± 7.89 ↑↑	0.53 ± 0.43
Il6	1.52 ± 0.98	36.44 ± 27.62 **	9.46 ± 7.14 ↑	1.84 ± 0.30	7.06 ± 12.44	0.48 ± 0.53
Nlrp3 (NALP3)	304.61 ± 32.31	577.15 ± 148.54 *	4.15 ± 1.85 ↑↑	1.93 ± 0.42 ↑↑	0.92 ± 0.27	0.78 ± 0.08 *
Nos2 (iNOS)	5.14 ± 4.14	54.03 ± 61.01 *	2362.11 ± 576.10 ↑↑	164.5 ± 29.20 ↑↑	1.76 ± 2.66	0.46 ± 0.60
Ptgs2 (COX-2)	4.04 ± 3.05	18.42 ± 18.88	17.45 ± 5.82 ↑↑	13.00 ± 3.37 ↑↑	11.28 ± 16.27	1.10 ± 0.98
Ptk2b (PYK2)	440.27 ± 71.46	708.42 ± 316.05	9.27 ± 0.81 ↑↑	5.32 ± 0.72 ↑↑	0.60 ± 0.19	0.48 ± 0.27 ↓
Pycard (ASC)	402.60 ± 181.20	999.91 ± 252.46 **	0.49 ± 0.07	0.44 ± 0.06 ↓*	0.99 ± 0.52	0.76 ± 0.16 *
Tnf (TNF-α)	126.44 ± 52.12	181.49 ± 135.49	4.93 ± 1.81 ↑↑	3.83 ± 1.20 ↑↑	0.62 ± 0.13	0.59 ± 0.33
Tnfrsf1a (TNFR1)	323.53 ± 22.84	348.26 ± 58.06	2.68 ± 0.34 ↑↑	2.88 ± 0.24 ↑↑	0.66 ± 0.06 ↓↓	0.78 ± 0.17 ↓
Tnfrsf1b (TNFR2)	304.18 ± 101.46	312.34 ± 128.06	2.37 ± 0.64 ↑	1.77 ± 0.41	0.40 ± 0.15 ↓↓	0.53 ± 0.28

Sex- and development-dependent responses of rat microglia to pro- and anti-inflammatory stimulation

Starlee Lively, Raymond Wong, Doris Lam, and Lyanne C. Schlichter
Correspondence: Professor Lyanne C. Schlichter Lyanne.Schlichter@uhnresearch.ca or Dr. Starlee Lively Starlee.Lively@uhnresearch.ca

Supplementary Table 11. Age comparisons – Female. Transcript expression of anti-inflammatory genes and receptors

Treatments, data presentation and analysis were as in Supplementary Table 10.

	Control		I+T		IL-4	
	mRNA counts ± SD		*Fold change with respect to Control± SD*			
	P1	P21	P1	P21	P1	P21
Arg1	2.48 ± 1.75	8.21 ± 6.62	5.19 ± 2.35 ↑	1.18 ± 1.35	4.83 ± 2.33 ↑↑	1.48 ± 2.22
Ccl22	3.48 ± 2.77	30.51 ± 12.61 **	3.47 ± 1.52	1.66 ± 0.85	35.21 ± 50.74 ↑↑	1.31 ± 1.40
Cd163	3.46 ± 2.80	26.99 ± 33.07 *	0.85 ± 0.64	0.37 ± 0.15	1.66 ± 0.89	2.43 ± 1.55 **
IL1rn (IL-RA)	638.08 ± 606.75	566.29 ± 192.81	4.58 ± 2.08 ↑↑	6.14 ± 2.49 ↑↑	0.27 ± 0.08	0.70 ± 0.29
Il4	4.04 ± 3.40	3.26 ± 1.68	0.41 ± 0.20	1.56 ± 0.77	0.71 ± 0.67	0.87 ± 0.59
Il4r (IL-4RA)	173.03 ± 15.26	216.28 ± 69.25	4.31 ± 1.65 ↑↑	2.28 ± 0.40 ↑↑	1.06 ± 0.47	0.96 ± 0.10
Il10	2.00 ± 1.65	6.46 ± 6.17	0.53 ± 0.05	0.59 ± 0.54	0.59 ± 0.22	0.56 ± 0.52
Il10ra	196.93 ± 42.39	293.83 ± 76.10 *	3.98 ± 0.70 ↑↑	2.80 ± 0.61 ↑↑	0.70 ± 0.17	0.87 ± 0.15 **
Il10rb	369.42 ± 30.06	361.31 ± 71.64	1.34 ± 0.24	1.44 ± 0.11 ↑↑	0.50 ± 0.09 ↓↓	0.67 ± 0.10 ↓↓
Il13	2.35 ± 2.43	3.41 ± 1.47	0.63 ± 0.31	1.12 ± 0.90	0.54 ± 0.19	0.60 ± 0.35
Il13ra1	147.35 ± 19.28	208.67 ± 105.89	2.55 ± 0.38 ↑↑	1.22 ± 0.30	0.55 ± 0.10 ↓	0.34 ± 0.11 ↓↓
Mrc1 (CD206)	275.30 ± 52.32 **	104.40 ± 83.46	0.01 ± 0.01 ↓↓	0.02 ± 0.01 ↓↓	2.46 ± 0.65 ↑ **	1.65 ± 0.56
Pparg	68.73 ± 62.18 **	3.41 ± 1.47	0.06 ± 0.04 ↓↓	1.50 ± 0.91	0.79 ± 0.64 **	1.43 ± 0.78
Pprc1	43.46 ± 9.42	62.75 ± 17.69	0.86 ± 0.32	1.09 ± 0.08	1.13 ± 0.27	1.12 ± 0.33
Retnla (FIZZ1)	5.05 ± 4.58	3.92 ± 1.31	0.26 ± 0.11	0.89 ± 0.41	0.47 ± 0.27	0.68 ± 0.26
Tgfb1	3223.42 ± 1199.60	3349.10 ± 856.59	0.53 ± 0.11 ↓	0.54 ± 0.07 ↓	0.90 ± 0.25	1.12 ± 0.21
Tgfbr1	1413.98 ± 260.64	1723.32 ± 703.82	0.51 ± 0.06 ↓	0.55 ± 0.15	0.42 ± 0.08 ↓↓	0.51 ± 0.25 ↓↓
Tgfbr2	409.96 ± 51.64	446.37 ± 129.05	1.32 ± 0.31	1.23 ± 0.18	0.52 ± 0.12 ↓↓	0.65 ± 0.11 ↓

Sex- and development-dependent responses of rat microglia to pro- and anti-inflammatory stimulation

Starlee Lively, Raymond Wong, Doris Lam, and Lyanne C. Schlichter
Correspondence: Professor Lyanne C. Schlichter Lyanne.Schlichter@uhnresearch.ca or Dr. Starlee Lively Starlee.Lively@uhnresearch.ca

Supplementary Table 12. Age comparisons – Female. Transcript expression of selected microglia markers and immune modulators

Treatments, data presentation and analysis were as in Supplementary Table 10.

	Control		I+T		IL-4	
	mRNA counts ± SD		*Fold change with respect to Control± SD*			
	P1	P21	P1	P21	P1	P21
Aif (Iba1)	5516.37 ± 1240.64	6189.06 ± 2004.63	1.77 ± 0.38	1.66 ± 0.20	0.61 ± 0.26 ↓	0.59 ± 0.31 ↓
Cd200r1	685.14 ± 358.04	516.57 ± 220.77	0.08 ± 0.02 ↓↓	0.17 ± 0.03 ↓↓	0.53 ± 0.19	1.05 ± 0.28
Cd68	12684.43 ± 2405.68 **	4102.14 ± 789.86	0.53 ± 0.13 ↓↓	1.24 ± 0.19	0.48 ± 0.13 ↓↓ **	0.82 ± 0.13
Csf1	678.53 ± 277.96	438.90 ± 485.42	0.47 ± 0.12	0.34 ± 0.10	1.67 ± 0.77	2.65 ± 1.44 ↑
Csf1r	2439.41 ± 528.86	3538.54 ± 935.12	0.79 ± 0.19	0.47 ± 0.06 ↓↓	0.88 ± 0.26	0.70 ± 0.14
Cx3cr1	329.29 ± 131.31	2173.62 ± 1064.17 **	0.02 ± 0.02 ↓↓	0.16 ± 0.10 ↓↓ **	0.43 ± 0.31	0.45 ± 0.46 **
F2r (PAR-1)	9.10 ± 5.20	18.75 ± 28.62	0.96 ± 0.41	0.21 ± 0.07	12.01 ± 9.62 ↑↑	2.43 ± 1.67
Hmox1 (HO-1)	1738.47 ± 568.59	3110.59 ± 988.14	3.58 ± 1.31 ↑↑	1.95 ± 0.46	0.67 ± 0.43	0.58 ± 0.28
Itgam (CD11b)	1010.94 ± 479.73	3438.99 ± 689.03 **	1.43 ± 0.20	1.32 ± 0.22 **	0.53 ± 0.32	0.63 ± 0.22 **
Lcn2 (NGAL)	6.03 ± 3.53	114.52 ± 125.20 **	1.43 ± 0.27	0.60 ± 0.42	3.46 ± 4.93	0.47 ± 0.34 *
Nfe2l2 (NRF2)	1004.03 ± 198.64	1201.70 ± 513.10	2.21 ± 0.33 ↑↑	1.84 ± 0.18 ↑↑	0.63 ± 0.13	0.54 ± 0.18 ↓
Nfkbia (IκBα)	1153.23 ± 232.75	3498.0 ± 1078.69 **	5.99 ± 0.85 ↑↑	2.04 ± 0.19 ↑↑	0.75 ± 0.32	0.57 ± 0.23 ↓ **
Nr3c1 (GR)	255.16 ± 20.84	318.70 ± 61.25	2.78 ± 0.34 ↑↑	2.47 ± 0.53 ↑↑	0.65 ± 0.08 ↓↓ **	0.90 ± 0.14
Pdcd1 (PD-1)	4.24 ± 2.87	4.53 ± 1.87	1.44 ± 0.66	0.91 ± 0.72	0.66 ± 0.35	0.97 ± 1.29
Tfrc (CD71)	284.72 ± 99.61 **	136.33 ± 48.56	0.64 ± 0.09	0.34 ± 0.09 ↓↓**	0.97 ± 0.26	1.33 ± 0.38
Tlr2	964.98 ± 142.28	2630.18 ± 1227.00	1.90 ± 0.76	1.42 ± 0.12	0.27 ± 0.11 ↓↓	0.29 ± 0.30 ↓↓
Tlr4	169.10 ± 41.73	268.77 ± 160.08	0.56 ± 0.13	0.50 ± 0.11	1.88 ± 0.37 ↑	1.51 ± 0.61
Trem2	2994.71 ± 569.13 **	1015.54 ± 244.49	0.04 ± 0.01 ↓↓	0.10 ± 0.05 ↓↓	0.27 ± 0.08 ↓↓	0.80 ± 0.24

Sex- and development-dependent responses of rat microglia to pro- and anti-inflammatory stimulation

Starlee Lively, Raymond Wong, Doris Lam, and Lyanne C. Schlichter
Correspondence: Professor Lyanne C. Schlichter Lyanne.Schlichter@uhnresearch.ca or Dr. Starlee Lively Starlee.Lively@uhnresearch.ca

Supplementary Table 13. Age comparisons – Female. Transcript expression of genes related to microglial physiology

Treatments, data presentation and analysis were as in Supplementary Table 10.

	Control			I+T			IL-4	
	mRNA counts ± SD			*Fold change with respect to Control± SD*				
	P1	P21		P1	P21		P1	P21
Cybb (NOX2)	473.38 ± 240.81	305.02 ± 116.77		2.22 ± 0.46 ↑	1.80 ± 0.27		0.35 ± 0.16 ↓	0.45 ± 0.35
Kcna3 (Kv1.3)	14.06 ± 5.90	56.29 ± 24.24 **		3.09 ± 1.29 ↑	2.62 ± 0.98 ↑ **		1.23 ± 0.29	0.59 ± 0.24
Kcna5 (Kv1.5)	3.79 ± 3.62	4.26 ± 2.24		0.28 ± 0.03	0.66 ± 0.19		0.53 ± 0.38	0.59 ± 0.42
Kcnj2 (Kir2.1)	443.00 ± 173.46	589.24 ± 268.80		5.28 ± 1.26 ↑↑	4.86 ± 1.11 ↑↑		0.30 ± 0.05 ↓↓	0.47 ± 0.16 ↓
Kcnn3 (KCa2.3)	3.98 ± 2.91	3.26 ± 1.68		1.14 ± 0.71	3.69 ± 3.31		0.53 ± 0.25	0.63 ± 0.37
Kcnn4 (KCa3.1)	14.16 ± 3.52	16.30 ± 12.76		1.97 ± 1.14	1.50 ± 0.54		1.79 ± 1.47	1.17 ± 0.64
Mmp9	237.88 ± 131.87	133.14 ± 93.77		0.75 ± 0.48	0.83 ± 0.42		2.43 ± 2.23	1.16 ± 0.80
Msr1 (SR-A)	2718.76 ± 1239.87	3783.79 ± 1373.80		0.27 ± 0.08 ↓↓ *	0.41 ± 0.13 ↓		0.38 ± 0.15 ↓↓ **	0.67 ± 0.23
Ncf1	1710.31 ± 294.93	4949.72 ± 2269.11		4.97 ± 1.71 ↑↑	1.91 ± 0.63 **		0.43 ± 0.10 ↓	0.44 ± 0.31 ↓ **
P2rx4	885.24 ± 108.07	1191.96 ± 570.22		2.91 ± 0.88 ↑↑	1.22 ± 0.52		0.59 ± 0.12	0.78 ± 0.29
P2rx7	54.73 ± 12.89	489.79 ± 110.07 **		3.48 ± 1.49 ↑↑	0.56 ± 0.17		1.43 ± 0.47	0.94 ± 0.15 **
P2ry2	45.70 ± 17.15	45.67 ± 9.92		2.58 ± 0.63 ↑↑	1.43 ± 0.25		1.82 ± 1.00	1.11 ± 0.49
P2ry12	131.18 ± 53.30	304.16 ± 138.63		0.36 ± 0.16 ↓	0.16 ± 0.06 ↓↓		1.44 ± 0.97	0.73 ± 0.30

Sex- and development-dependent responses of rat microglia to pro- and anti-inflammatory stimulation

Starlee Lively, Raymond Wong, Doris Lam, and Lyanne C. Schlichter
Correspondence: Professor Lyanne C. Schlichter Lyanne.Schlichter@uhnresearch.ca or Dr. Starlee Lively Starlee.Lively@uhnresearch.ca

Supplementary Table 14. Age comparisons – Male. Expression of pro-inflammatory mediators
Microglia were isolated from 1- and 21-day-old male rat pups and stimulated with IFN-γ + TNF-α (I+T), IL-4 or IL-10 for 24 h. For clarity, protein names are included for some genes. To show differences in basal mRNA levels, unstimulated (control) counts are expressed as mean counts/100 ng RNA sample ± SD (n=4–7 individual cultures). Effects of activation state on a given gene are expressed as fold-changes relative to age-matched control levels; arrows represent significant increases (red arrows) or decreases (blue arrows) in expression. Age differences within an activation paradigm are indicated by asterisks. One symbol (arrow or asterisk) indicates p<0.05; two, p<0.01; three, p<0.001.

	Control		I+T		IL-4	
	mRNA counts ± SD		*Fold change with respect to Control± SD*			
	P1	P21	P1	P21	P1	P21
Casp1 (ICE)	133.94 ± 23.39	260.58 ± 100.78 *	2.06 ± 0.09 ↑↑	2.23 ± 0.45 ↑↑ **	0.88 ± 0.22	0.73 ± 0.22
Cd274 (PD-L1)	159.36 ± 37.91	274.88 ± 120.07	12.89 ± 2.04 ↑↑	4.78 ± 1.22 ↑↑	2.60 ± 0.98 ↑	1.18 ± 0.68
Cxcl10 (IP-10)	147.95 ± 79.76	767.39 ± 671.94	38.19 ± 5.27 ↑↑	16.99 ± 1.48 ↑↑	1.52 ± 1.47	0.25 ± 0.32
Ifnb1	1.76 ± 1.50	1.79 ± 0.10	3.37 ± 3.17	6.95 ± 3.81 ↑	2.79 ± 3.61	0.95 ± 0.37
Ifng	1.20 ± 0.26	1.79 ± 0.10	0.89 ± 0.15	1.16 ± 0.29	1.48 ± 1.23	0.86 ± 0.18
Ifngr1	2813.78 ± 390.78	1668.98 ± 706.10	0.54 ± 0.13 ↓	0.46 ± 0.02 ↓ *	0.36 ± 0.11 ↓↓	0.80 ± 0.41
Ifngr2	6.35 ± 3.78	28.68 ± 9.18 **	1.63 ± 0.55	1.43 ± 0.52 **	1.83 ± 0.86	0.84 ± 0.45
Il1a	81.58 ± 45.03	694.22 ± 668.32 *	5.89 ± 1.94 ↑	0.63 ± 0.43	5.26 ± 4.51 ↑	0.37 ± 0.21
Il1b	173.51 ± 91.79	1853.48 ± 1597.34 *	4.61 ± 1.55	1.03 ± 0.25	1.48 ± 1.33	0.21 ± 0.18
Il1r1	1.98 ± 1.33	13.27 ± 14.55	2.80 ± 1.71	1.26 ± 0.94	2.38 ± 2.66	0.79 ± 0.82
Il1r2	1.16 ± 0.17	12.02 ± 11.13 **	5.10 ± 4.41 ↑	0.90 ± 0.33	7.75 ± 5.93 ↑	0.55 ± 0.25
Il6	4.36 ± 3.81	59.63 ± 60.03 *	2.86 ± 1.58	1.90 ± 1.16 *	1.30 ± 1.52	0.25 ± 0.16
Nlrp3 (NALP3)	296.58 ± 81.78	584.05 ± 186.45	4.13 ± 1.39 ↑↑	1.65 ± 0.26	1.40 ± 0.69	0.81 ± 0.23
Nos2 (iNOS)	3.80 ± 4.06	91.09 ± 97.83 **	3081.04 ± 486.77 ↑↑	103.04 ± 62.47 ↑↑	3.28 ± 3.72	0.15 ± 0.08
Ptgs2 (COX-2)	5.52 ± 5.84	39.17 ± 33.05	17.80 ± 2.91 ↑↑	5.68 ± 0.91	8.37 ± 12.10	0.28 ± 0.08
Ptk2b (PYK2)	376.19 ±36.59	662.25 ± 294.29	10.73 ± 1.18 ↑↑	5.07 ± 0.58 ↑↑	0.80 ± 0.31	0.53 ± 0.27
Pycard (ASC)	385.83 ± 90.48	776.71 ± 293.66 *	0.50 ± 0.04 ↓	0.60 ± 0.12 **	1.22 ± 0.42	1.04 ± 0.20 *
Tnf (TNF-α)	121.67 ± 85.27	252.09 ± 209.53	4.91 ± 1.14 ↑↑	2.92 ± 0.47 ↑	1.05 ± 0.52	0.34 ± 0.16
Tnfrsf1a (TNFR1)	291.94 ± 60.43	334.12 ± 83.03	2.88 ± 0.17 ↑↑	3.01 ± 0.34 ↑↑	0.78 ± 0.07	0.78 ± 0.11
Tnfrsf1b (TNFR2)	272.41 ± 55.11	316.13 ± 151.47	2.38 ± 0.48 ↑	1.75 ± 0.55	0.47 ± 0.16 ↓	0.52 ± 0.28

Sex- and development-dependent responses of rat microglia to pro- and anti-inflammatory stimulation

Starlee Lively, Raymond Wong, Doris Lam, and Lyanne C. Schlichter
Correspondence: Professor Lyanne C. Schlichter Lyanne.Schlichter@uhnresearch.ca or Dr. Starlee Lively Starlee.Lively@uhnresearch.ca

Supplementary Table 15. Age comparisons – Male. Transcript expression of anti-inflammatory genes and receptors
Treatments, data presentation and analysis were as in Supplementary Table 14.

	Control		I+T		IL-4	
	mRNA counts ± SD		*Fold change with respect to Control± SD*			
	P1	P21	P1	P21	P1	P21
Arg1	1.39 ± 0.42	1.79 ± 0.10	3.39 ± 1.24 ↑	6.51 ± 1.05 ↑↑	10.44 ± 9.17 ↑↑	3.61 ± 1.74
Ccl22	1.39 ± 0.40	46.90 ± 38.42 **	4.33 ± 2.29	0.79 ± 0.68	133.27 ± 201.19 ↑↑	0.32 ± 0.12
Cd163	3.23 ± 2.96	40.84 ± 61.65 *	1.47 ± 0.77	0.12 ± 0.08	1.56 ± 0.81	1.34 ± 0.95 *
IL1rn (IL-RA)	267.87 ±186.87	531.84 ± 251.10	8.37 ± 1.62 ↑↑	5.86 ± 1.87 ↑↑	0.75 ± 0.53	0.63 ± 0.29
Il4	2.04 ± 2.13	2.41 ± 1.33	1.61 ± 1.40	1.53 ± 0.61	1.79 ± 3.15	0.82 ± 0.32
Il4r (IL-4RA)	175.25 ± 42.99	281.60 ± 138.03	3.84 ± 0.76 ↑↑	1.90 ± 0.44 ↑	1.13 ± 0.44	0.86 ± 0.23
Il10	1.20 ± 0.15	5.76 ± 6.54 *	0.91 ± 0.15	0.53 ± 0.42	1.00 ± 0.34	0.27 ± 0.06
Il10ra	167.93 ± 22.72	261.00 ± 84.76	4.59 ± 0.54 ↑↑	2.87 ± 0.65 ↑↑	0.96 ± 0.33	0.96 ± 0.19
Il10rb	340.12 ± 16.43	328.22 ± 107.51	1.50 ± 0.18	1.51 ± 0.21	0.55 ± 0.12 ↓↓	0.73 ± 0.17
Il13	1.48 ± 0.87	2.07 ± 0.58	0.99 ± 0.63	1.49 ± 1.18	1.67 ± 2.22	0.74 ± 0.16
Il13ra1	138.07 ± 18.85	176.16 ± 85.03	2.63 ± 0.26 ↑↑	1.39 ± 0.24	0.55 ± 0.15	0.50 ± 0.27
Mrc1 (CD206)	282.90 ± 40.27 *	99.25 ± 68.65	0.01 ± 0.01 ↓↓	0.02 ± 0.01 ↓↓	2.28 ± 0.65 **	1.47 ± 0.80
Pparg	23.98 ± 17.35 *	3.37 ± 1.61	0.17 ± 0.07 ↓	0.61 ± 0.16	1.67 ± 1.07 **	0.59 ± 0.26
Pprc1	43.12 ± 8.03	70.44 ± 27.48	0.95 ± 0.12	0.94 ± 0.12	1.58 ± 0.44	0.98 ± 0.17
Retnla (FIZZ1)	1.39 ± 0.47	2.04 ± 0.60	1.93 ± 1.22	1.87 ± 1.79	2.46 ± 3.61	1.13 ± 0.37
Tgfb1	3007.48 ± 1278.53	3134.67 ± 810.30	0.60 ± 0.05	0.59 ± 0.05	1.10 ± 0.37	1.02 ± 0.11
Tgfbr1	1446.21 ± 242.81	1386.49 ± 475.92	0.48 ± 0.05 ↓↓	0.73 ± 0.22	0.43 ± 0.09 ↓↓	0.65 ± 0.36
Tgfbr2	412.76 ± 51.04	444.76 ± 184.83	1.19 ± 0.09	1.31 ± 0.21	0.49 ± 0.11 ↓↓	0.60 ± 0.12

Sex- and development-dependent responses of rat microglia to pro- and anti-inflammatory stimulation

Starlee Lively, Raymond Wong, Doris Lam, and Lyanne C. Schlichter
Correspondence: Professor Lyanne C. Schlichter Lyanne.Schlichter@uhnresearch.ca or Dr. Starlee Lively Starlee.Lively@uhnresearch.ca

Supplementary Table 16. Age comparisons – Male. Transcript expression of selected microglia markers and immune modulators

Treatments, data presentation and analysis were as in Supplementary Table 14.

	Control		I+T		IL-4	
	mRNA counts ± SD		*Fold change with respect to Control± SD*			
	P1	P21	P1	P21	P1	P21
Aif (Iba1)	5204.37 ± 534.70	5489.12 ± 1946.66	1.88 ± 0.35 ↑	1.74 ± 0.29	0.59 ± 0.23 ↓	0.78 ± 0.49
Cd200r1	640.20 ± 412.42	457.71 ± 144.96	0.08 ± 0.01 ↓↓	0.24 ± 0.13 ↓↓	0.64 ± 0.27	0.98 ± 0.23
Cd68	12213.24 ± 1695.55 **	3365.60 ± 726.52	0.60 ± 0.10 ↓↓ *	1.37 ± 0.21	0.49 ± 0.09 ↓↓ **	0.88 ± 0.28
Csf1	650.03 ± 257.69	481.21 ± 567.72	0.39 ± 0.06	0.49 ± 0.33	2.10 ± 0.78	1.67 ± 0.93
Csf1r	2190.31 ± 290.70	3334.08 ± 748.76 *	0.80 ± 0.12	0.53 ± 0.04 ↓↓	0.89 ± 0.16	0.82 ± 0.16 *
Cx3cr1	425.55 ± 153.49	1693.39 ± 811.42 *	0.01 ± 0.01 ↓↓	0.19 ± 0.09 ↓ **	0.31 ± 0.22 ↓	0.74 ± 0.63 **
F2r (PAR-1)	15.17 ± 14.62	35.03 ± 40.67	0.35 ± 0.15	0.18 ± 0.17	8.53 ± 12.54 ↑	0.94 ± 0.56
Hmox1 (HO-1)	1703.47 ± 226.05	2501.47 ± 1409.96	3.90 ± 0.70 ↑↑	2.26 ± 0.69 ↑	0.72 ± 0.49	0.58 ± 0.23
Lcn2 (NGAL)	7.24 ± 5.92	192.18 ± 139.37 **	1.16 ± 0.40	1.55 ± 2.54 *	1.06 ± 1.15	0.31 ± 0.22 *
Itgam (CD11b)	948.16 ± 459.69	3439.18 ± 1016.25 **	1.55 ± 0.25	1.46 ± 0.32 **	0.61 ± 0.39	0.67 ± 0.41 **
Nfe2l2 (NRF2)	825.22 ± 96.50	1150.79 ± 416.04	2.64 ± 0.30 ↑↑	1.95 ± 0.42 ↑	0.95 ± 0.28	0.60 ± 0.34
Nfkbia (IκBα)	1149.76 ± 254.18	3279.54 ± 1376.99 *	6.17 ± 0.92 ↑↑	2.13 ± 0.16	1.27 ± 0.71	0.59 ± 0.29
Nr3c1 (GR)	263.36 ± 11.73	306.02 ± 56.71	2.76 ± 0.56 ↑↑	2.00 ± 0.36 ↑↑	0.72 ± 0.14 ↓ **	0.93 ± 0.12
Pdcd1 (PD-1)	3.13 ± 2.05	2.29 ± 0.76	1.56 ± 1.04	3.11 ± 3.17	0.94 ± 1.03	1.46 ± 1.55
Tfrc (CD71)	278.90 ± 69.04	168.68 ± 75.83	0.79 ± 0.22	0.44 ± 0.47 ↓ **	1.26 ± 0.25	1.09 ± 0.34
Tlr2	1030.66 ± 255.52	2743.05 ± 1404.66	1.91 ± 0.47	1.34 ± 0.10	0.29 ± 0.13 ↓↓	0.34 ± 0.35 ↓
Tlr4	166.97 ± 49.87	245.04 ± 123.10	0.60 ± 0.15	0.50 ± 0.12	2.05 ± 0.66 ↑	1.40 ± 0.55
Trem2	2967.91 ± 614.30 *	950.48 ± 199.99	0.04 ± 0.03 ↓↓	0.18 ± 0.17 ↓↓	0.27 ± 0.12 ↓↓	0.89 ± 0.31

Sex- and development-dependent responses of rat microglia to pro- and anti-inflammatory stimulation

Starlee Lively, Raymond Wong, Doris Lam, and Lyanne C. Schlichter
Correspondence: Professor Lyanne C. Schlichter Lyanne.Schlichter@uhnresearch.ca or Dr. Starlee Lively Starlee.Lively@uhnresearch.ca

Supplementary Table 17. Age comparisons – Male. Transcript expression of genes related to microglial physiology

Treatments, data presentation and analysis were as in Supplementary Table 14.

	Control		I+T		IL-4	
	mRNA counts ± SD		*Fold change with respect to Control± SD*			
	P1	P21	P1	P21	P1	P21
Cybb (NOX2)	411.69 ± 145.20	305.07 ± 163.21	2.36 ± 0.31	1.89 ± 0.18	0.37 ± 0.19 ↓	0.52 ± 0.48
Kcna3 (Kv1.3)	17.20 ± 6.79	56.48 ± 27.96 **	2.59 ± 0.83 ↑↑	3.41 ± 1.44 ↑↑ **	0.94 ± 0.29	0.65 ± 0.18 *
Kcna5 (Kv1.5)	1.48 ± 0.87	3.38 ± 2.96	1.07 ± 0.61	0.91 ± 0.72	1.53 ± 1.87	0.66 ± 0.49
Kcnj2 (Kir2.1)	414.86 ± 238.40	585.97 ± 372.06	5.02 ± 0.63 ↑↑	4.63 ± 1.41 ↑↑	0.41 ± 0.09	0.53 ± 0.38
Kcnn3 (KCa2.3)	1.20 ± 0.26	3.17 ± 2.51	2.30 ± 1.79	1.99 ± 1.12	1.00 ± 0.34	0.53 ± 0.21
Kcnn4 (KCa3.1)	9.04 ± 5.96	15.99 ± 12.56	2.27 ± 0.87	1.71 ± 0.80	2.79 ± 2.28	0.75 ± 0.12
Mmp9	293.77 ± 256.16	143.02 ± 47.74	0.29 ± 0.10	1.10 ± 0.63	2.23 ± 2.23	0.66 ± 0.28
Msr1 (SR-A)	2452.06 ± 1587.45	4193.33 ± 1837.95	0.31 ± 0.12 ↓	0.38 ± 0.10	0.42 ± 0.2	0.57 ± 0.22
Ncf1	1844.46 ± 518.70	3785.28 ± 1476.39	5.60 ± 2.20 ↑↑	2.52 ± 0.74 ↑	0.51 ± 0.19	0.60 ± 0.40
P2rx4	939.01 ± 292.71	764.61 ± 164.22	2.77 ± 0.54 ↑↑	1.98 ± 1.05	0.66 ± 0.15	1.21 ± 0.49
P2rx7	44.04 ± 16.93	383.92 ± 28.89 **	4.13 ± 2.57 ↑↑	0.57 ± 0.06	2.23 ± 0.58 ↑↑	1.06 ± 0.15 **
P2ry2	39.07 ± 27.04	49.90 ± 19.70	2.65 ± 0.75 ↑	1.24 ± 0.34	2.12 ± 1.26	0.73 ± 0.52
P2ry12	129.33 ± 48.62	238.47 ± 125.38	0.33 ± 0.12 ↓	0.24 ± 0.09 ↓	1.25 ± 1.15	1.20 ± 0.36
